# Noninvasive Detection and Subtyping of Esophageal Cancer by Circulating Cell‐Free mtDNA Fragmentomics: A Multi‐Center Validation Study

**DOI:** 10.1002/advs.78377

**Published:** 2026-10-28

**Authors:** Yue Liu, Wenying Yang, Yu Li, Siyuan Wang, Xiang Li, Fan Peng, Miao Dang, Huanqin Zhang, Huanmin Jiao, Yuqing Yang, Zhaoquan Su, Xiumin Ma, Zhiyun Gong, Renquan Lu, Jinliang Xing, Yang Liu

**Affiliations:** ^1^ State Key Laboratory of Holistic Integrative Management of Gastrointestinal Cancers and Department of Physiology and Pathophysiology Fourth Military Medical University Xi'an China; ^2^ College of Medical Technology Shaanxi University of Chinese Medicine Xianyang China; ^3^ Department of Clinical Laboratory The First Affiliated Hospital of Air Force Medical University Xi'an China; ^4^ Clinical Laboratory Center Tumor Hospital Affiliated to Xinjiang Medical University Urumqi China; ^5^ Department of Clinical Laboratory Fudan University Shanghai Cancer Center Shanghai China; ^6^ State Key Laboratory of Holistic Integrative Management of Gastrointestinal Cancers and Department of Clinical Diagnosis Tangdu Hospital Fourth Military Medical University Xi'an China

**Keywords:** circulating cell‐free mitochondrial DNA, early detection, esophageal cancer, fragmentomics

## Abstract

Esophageal cancer (EC) is highly lethal due to late diagnosis, and accurate non‐invasive tools for early detection and subtyping remain lacking. Circulating cell‐free mitochondrial DNA (ccf‐mtDNA) fragmentomics offers a promising biomarker source. In this large multi‐center study enrolling 1,260 participants spanning healthy control (HC), benign esophageal diseases (BED), precancerous lesions and EC patients, we developed two machine learning models based on plasma ccf‐mtDNA fragmentomic profiles: an EC detection (ED) model and a pathological subtyping prediction (PSP) model. The ED model achieved an AUC of 0.9706 (EC vs. non‐EC) in the training cohort, with high performance in internal (0.9762), external (0.9748), and Chinese minority (0.9668, Uyghur and Kazakh) validation cohorts. At the optimal cut‐off, it yielded 92.16% sensitivity for stage I EC in the combined validation cohort and correctly identified 93.10% of early EC cases missed by preoperative biopsy. It also discriminated high‐grade intraepithelial neoplasia from non‐EC with an AUC of 0.9597. The PSP model distinguished ESCC from EAC with 89.71% accuracy, improving to 90.74% after integrating key clinical parameters. In summary, ccf‐mtDNA fragmentomics provides a robust, non‐invasive platform for early EC detection, precancerous risk stratification, and histological subtyping, demonstrating strong potential to transform clinical management.

## Graphical abstract

Circulating cell‐free mitochondrial DNA provides a robust, noninvasive platform for early detection and histological subtyping of esophageal cancer. This large multi‐center study validates consistently high accuracy across ethnically and geographically diverse cohorts, demonstrating strong potential to transform clinical management.

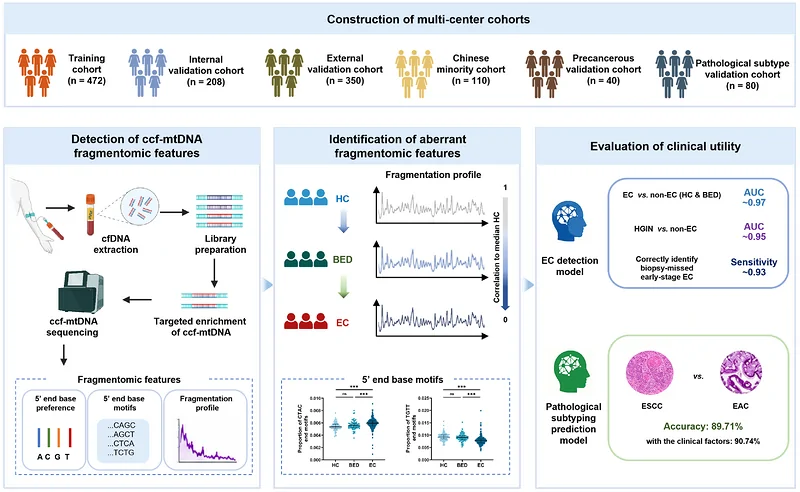

## 1 Introduction

Esophageal cancer (EC) remains a lethal malignancy, with an overall 5‐year survival of 15–25% [1, 2], largely because early‐stage EC is frequently asymptomatic, leading to delayed detection and diagnosis at advanced stages. While endoscopy effectively detects intraepithelial neoplasia (IEN) and early carcinomas in high‐risk regions, its invasiveness, cost, and limited accessibility pose significant barriers to population‐wide implementation. Consequently, the detection rate for early lesions remains critically low, particularly for high‐grade intraepithelial neoplasia (HGIN), which represents a critical target for early intervention to reduce mortality [3, 4]. There is therefore an urgent, unmet need for a non‐invasive, accurate, and accessible tool for the early detection of EC and its precancerous stages [5].

However, early detection of EC faces two fundamental challenges. First, available non‐invasive methods lack sufficient sensitivity. For example, serum biomarkers such as squamous cell carcinoma antigen (SCCA) for esophageal squamous cell carcinoma (ESCC) and Carcinoembryonic antigen (CEA) for esophageal adenocarcinoma (EAC), demonstrate unacceptably low sensitivity [6], typically below 30 percent for early‐stage EC. The second is the need for reliable histological subtyping (ESCC *vs*. EAC), which dictates distinct treatment pathways given their different origins and biology [7]. Currently, this differentiation remains dependent on invasive biopsy. A non‐invasive method that addresses both challenges would be transformative, paving the way for timely and personalized intervention.

The analysis of circulating cell‐free DNA (cfDNA) offers a promising non‐invasive approach. Despite their biological specificity, conventional cfDNA biomarkers lack early‐stage sensitivity due to low circulating tumor DNA (ctDNA) abundance and background noise from non‐malignant inflammation [8]. While emerging methods aim to overcome these limits, each presents distinct challenges. For instance, whole‐genome bisulfite sequencing (WGBS) of cfDNA methylomes has shown potential for ESCC detection [9], but the bisulfite conversion step can fragment and degrade already scarce cfDNA. Similarly, fragmentomic features derived from whole‐genome sequencing (WGS) data have been used in ensemble models for early ESCC detection [10]. However, the reproducibility of such fragmentation profiles remains uncertain, in part due to the technical constraints of low sequencing coverage, which may limit clinical translation.

In fact, plasma harbors circulating cell‐free mitochondrial DNA (ccf‐mtDNA), a small, high‐copy‐number genome (16,569 bp) that has emerged as a promising biomarker for cancer detection and monitoring via liquid biopsy [11, 12, 13, 14]. Notably, Li et al. have demonstrated that the downregulation of Mitochondrial Transcription Factor A (TFAM), a key mitochondrial transcription factor in esophageal cancer, directly induces mtDNA instability and its leakage into the cytoplasm, thereby driving tumor progression [15]. This core pathological process may alter the fragmentation patterns of ccf‐mtDNA and promote its release into the bloodstream, providing a rational basis for our fragmentomics‐based approach. We therefore hypothesize that high‐resolution fragmentomic analysis of plasma ccf‐mtDNA can capture biologically regulated, disease‐associated fragmentation patterns in circulation, offering a novel non‐invasive approach with high sensitivity and specificity for the early detection of EC. Technically, we have established a capture‐based sequencing method for ccf‐mtDNA that achieves greater depth and more comprehensive fragmentomic profiling than WGS [16]. Clinically, our prior studies have demonstrated the significant potential of cf‐mtDNA fragmentomics in early tumor detection and tissue‐of‐origin tracing, supporting its clinical utility in precision oncology [17, 18, 19].

To bridge the critical gap between biomarker discovery and clinical application, we developed and validated a unified diagnostic framework based on plasma ccf‑mtDNA fragmentomics. Using a large‑scale, multi‑center cohort encompassing geographically and ethnically diverse populations, our study aimed to address two pivotal clinical needs, one is the non‑invasive detection of HGIN and early‑stage EC to enable timely intervention, and the other is the accurate discrimination between ESCC and EAC to guide subtype‑specific treatment.

## 2 Methods

Detailed methods were described in the Supplementary Information.

## 3 Results

### 3.1 Study Design

To enable the noninvasive early detection and pathological subtyping of EC, we developed a machine learning framework utilizing plasma ccf‐mtDNA fragmentomics. This multi‐center study involved a total of 1,260 participants recruited across three clinical institutions. For development and validation of the ED model and evaluation of precancerous lesions, 720 participants were enrolled from Tangdu Hospital. Of these, 40 patients with low‐grade intraepithelial neoplasia (LGIN) or HGIN were designated as the precancerous validation cohort. And the 680 participants were randomly assigned to a training cohort for model development and an internal validation cohort for performance evaluation in a 7:3 ratio. Furthermore, we recruited an external validation cohort of 350 participants from Fudan University Shanghai Cancer Center and a Chinese minority cohort of 110 participants from Tumor Hospital Affiliated to Xinjiang Medical University. To further validate the performance of our EC subtype prediction model, we enrolled an independent cohort of 80 patients, including 40 ESCC and 40 EAC cases, from the three clinical institutions (Figure 1A).

**FIGURE 1:**
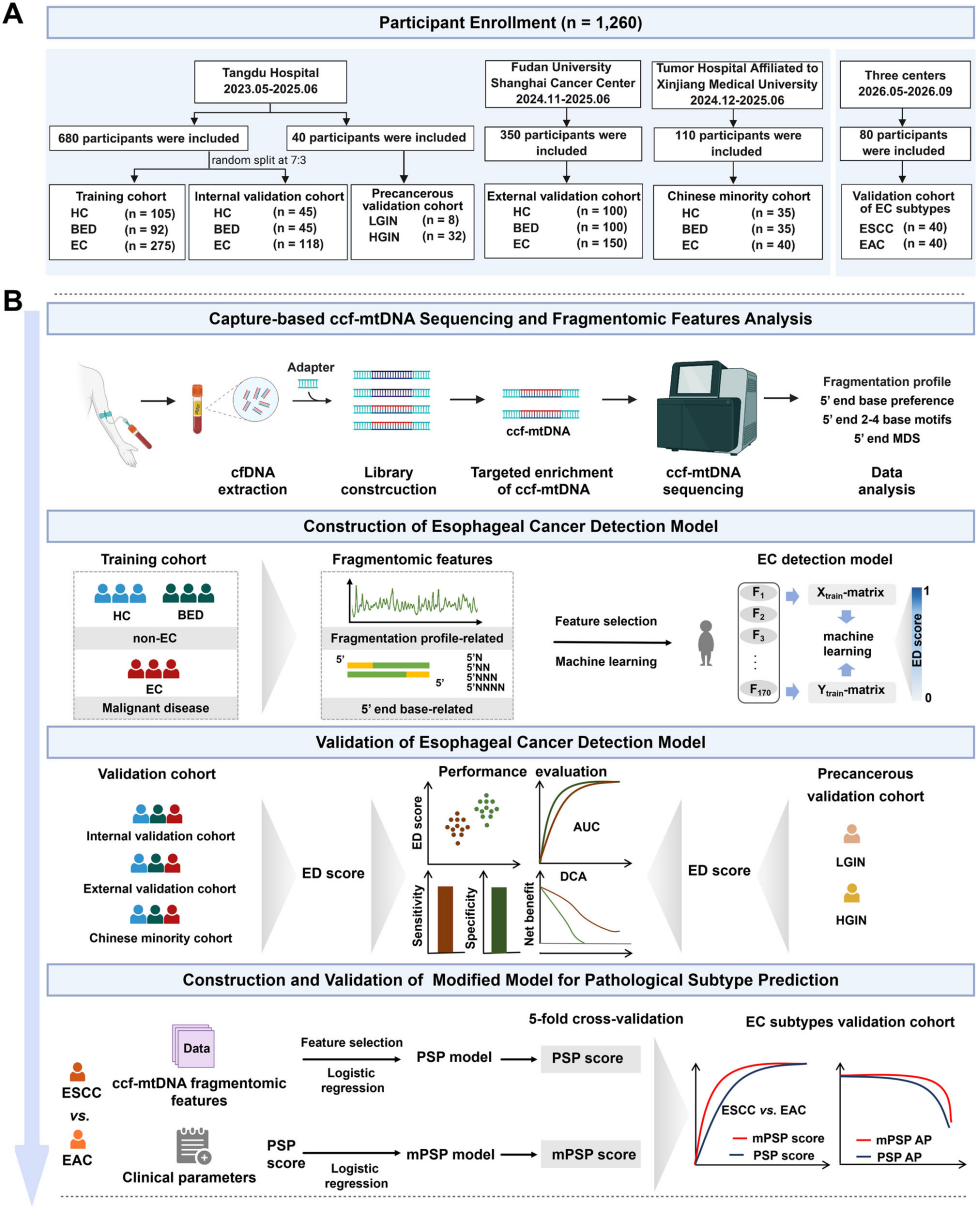
Study design and analytical framework for the detection of esophageal cancer and prediction of pathological subtypes based on ccf‐mtDNA fragmentomics. (A) Overview of participant enrollment and cohort allocation. A total of 1,260 participants from three centers (Tangdu Hospital, Fudan University Shanghai Cancer Center, and the Affiliated Tumor Hospital of Xinjiang Medical University) were enrolled. From Tangdu Hospital, 680 participants were randomly split at a 7:3 ratio into a training cohort and an internal validation cohort consisting of HC, BED, and EC patients, while 40 participants with LGIN or HGIN formed the precancerous cohort. Additionally, 350 participants from Fudan University Shanghai Cancer Center served as the external validation cohort, and 110 participants from Tumor Hospital Affiliated to Xinjiang Medical University formed the Chinese minority cohort to assess model generalizability. In addition, an independent multicenter cohort of 80 patients, including 40 patients with ESCC and 40 with EAC, was enrolled for independent validation of the pathological subtype prediction models. (B) Workflow for capture‐based ccf‐mtDNA sequencing and fragmentomic analysis. Plasma cfDNA was extracted, followed by library construction, targeted enrichment of mitochondrial DNA, and sequencing. Fragmentomic features, including fragmentation profiles, 5’ base preferences, end‐motif patterns, and fragment size distributions, were derived for downstream analyses. Using the training cohort, informative fragmentomic features were selected and integrated via a machine learning framework to construct an esophageal cancer detection model, which generated an ED score. The performance of the model was evaluated in the internal, external, and Chinese minority validation cohorts using ROC analysis and DCA. The ED score was further assessed in the precancerous cohort, including LGIN and HGIN. In addition, a modified model was developed for pathological subtype prediction between ESCC and EAC. ccf‐mtDNA fragmentomic features were subjected to feature selection and logistic regression to construct the PSP model, yielding PSP score. Clinical parameters were subsequently incorporated into a second logistic regression model together with the PSP score to derive a mPSP score. Model performance was evaluated using five‐fold cross‐validation. We then validated the performance of the PSP and mPSP models in an independent validation cohort. HC, healthy control; BED, benign esophageal diseases; EC, esophageal cancer; ESCC, esophageal squamous cell carcinoma; EAC, esophageal adenocarcinoma; LGIN, low‐grade intraepithelial neoplasia; HGIN, high‐grade intraepithelial neoplasia; cfDNA, cell‐free DNA; ccf‐mtDNA, circulating cell‐free mitochondrial DNA; ED, esophageal cancer detection; ROC, receiver operating characteristic; DCA, decision curve analysis; PSP, pathological subtype prediction; mPSP, modified pathological subtype prediction; PR, precision‐recall; AP, average precision.

Building upon these diverse cohorts, we implemented a systematic study workflow centered on plasma ccf‐mtDNA fragmentomics. This framework utilized capture‐based ccf‐mtDNA sequencing to generate fragmentomic features. These features were used to train a machine learning pipeline, first constructing an esophageal cancer detection (ED) model. Given the critical differences in therapy and prognosis between ESCC and EAC, we further developed a pathological subtyping prediction (PSP) strategy for refined classification (Figure 1B). Demographic, clinical, and lifestyle variables were summarized in Table S1. To control for potential confounders, age and sex were strictly matched across compared groups in all fragmentomic analyses (Figure S1).

### 3.2 EC Patients Exhibit Aberrant Plasma Ccf‐mtDNA Fragmentomic Profiles

We first asked whether patients with EC exhibit aberrant plasma ccf‐mtDNA fragmentomics compared with non‐EC. To investigate this, fragment size distribution (FSD) scores were calculated at single‐base resolution to characterize mtDNA fragmentation patterns, following previously established methodologies [17, 18, 19]. As shown in Figure 2A, the ccf‐mtDNA fragmentation profiles in EC patients were markedly more altered and heterogeneous than those in patients with BED and HC. Correlation analyses demonstrated a stepwise decrease in similarity to the median HC fragmentation profile from HC group to BED group, and further to EC group (all *p* < 0.05; Figure 2B), indicating progressive disruption of mtDNA fragmentation patterns during disease development. And then fifty representative EC‐associated features are shown in Figure 2C. Differential analysis identified significant bidirectional alterations in 697 features (51% of total features) between the EC and non‐EC (HC and BED) groups, as visualized by volcano plots (Figure 2D). Specifically, the proportions of TGTT‐ and TGT‐end motifs were significantly elevated in EC group, whereas those of CGAC‐end and CTAC‐end motifs were decreased (*p* < 0.05, Figure 2E–H), indicating EC‐specific fragmentomic features.

**FIGURE 2:**
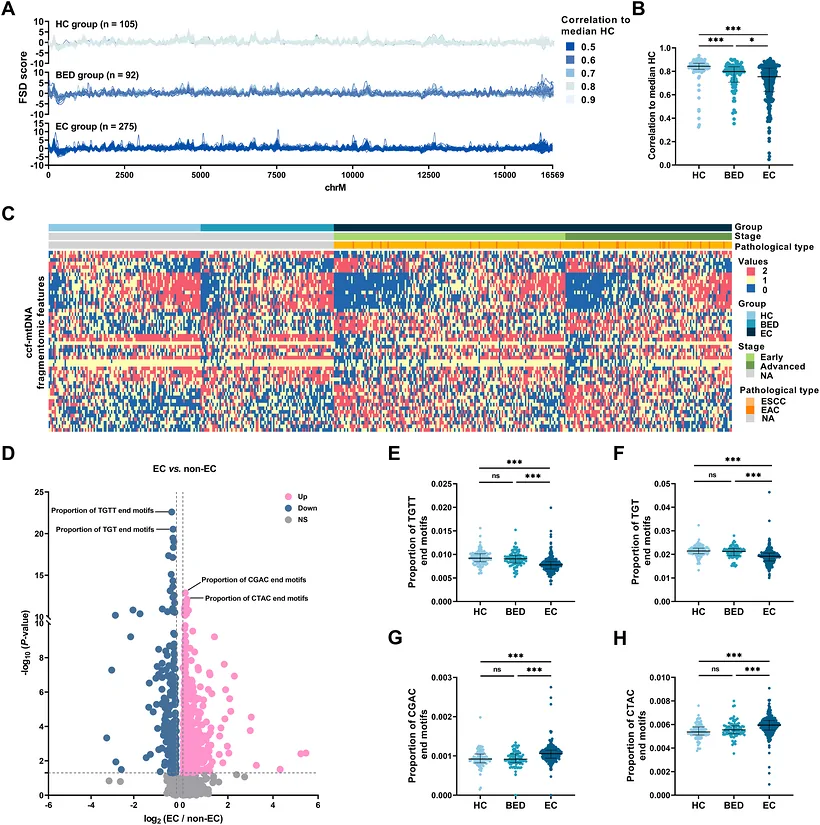
Comparison of plasma ccf‐mtDNA fragmentomic features in the training cohort. **(A**) ccf‐mtDNA fragmentation profiles for individual samples in the HC, BED, and EC groups, colored by correlation to the median HC profile. (B) Comparison of correlation coefficients between individual fragmentation profiles and the median HC profile within each group. (C) Heatmap showing the distribution of 50 selected fragmentomic features across subgroups. (D) Volcano plot of differential fragmentomic features between EC and non‐EC (HC and BED). Each point represents one feature; dashed lines indicate the thresholds used to define differential fragmentomic features; a positive log_2_ fold change indicates enrichment in EC. Comparison of (E) proportion of TGTT‐end motifs, (F) proportion of TGT‐end motifs, (G) proportion of CGAC‐end motifs, and (H) proportion of CTAC‐end motifs among HC, BED and EC groups. In panels B and E‐H, dots represent individual participants; horizontal lines indicate the median and IQR. ns, not significant; *, *p* < 0.05; ***, *p* < 0.001; HC, healthy control; BED, benign esophageal diseases; EC, esophageal cancer; ESCC, esophageal squamous cell carcinoma; EAC, esophageal adenocarcinoma; NA, Not Applicable; IQR, interquartile range.

To evaluate potential confounding, we assessed the effects of demographic and lifestyle factors, including age, sex, smoking, alcohol history, body mass index (BMI). Stratified analysis using principal component analysis (PCA) confirmed that samples from different subgroups overlapped significantly (Figures S2–S6), indicating that these factors did not substantially confound the fragmentomic profiles. Specifically, fragmentomic profiles showed no clustering by smoking, alcohol or BMI status within any clinical group (Figures S4–S6). Similarly, neither tumor location nor serum biomarker status significantly influenced the global fragmentation patterns (Figures S7 and S8). Although overall FSD scores did not differ significantly by tumor stage or size, differential feature analysis revealed stage‐ and size‐dependent alterations in specific fragmentomic features, as visualized in volcano plots (Figures S9 and S10). To further assess potential batch effects across the three participating centers, we performed principal component analysis stratified by clinical group (HC, BED, and EC). Within each group, samples from all centers showed substantial overlap without center‐specific clustering, indicating that inter‐center variation did not dominate the data structure (Figure S11). To gain further biological insight into these specific fragmentation patterns, we quantified the plasma levels of key nucleases. Compared with HC, the concentrations of deoxyribonuclease 1 (DNASE1), deoxyribonuclease 1‐like 3 (DNASE1L3), DNA fragmentation factor subunit beta (DFFB), and endonuclease G (ENDOG) were significantly altered in both the BED and EC groups (Figure S12). Furthermore, EAC and ESCC exhibited significantly different plasma concentrations of DNASE1L3, DNASE1, and DFFB (Figure S13). These findings demonstrate that esophageal lesions and histological subtypes are associated with distinct circulating nuclease profiles, providing a biologically plausible link to the observed alterations in ccf‐mtDNA fragmentation. In summary, these results confirm that EC is characterized by distinct plasma ccf‐mtDNA fragmentomic features, supporting their utility for developing a detection model.

### 3.3 The ED Model Maintains High Performance in Independent and Ethnically Diverse Cohorts

To translate ccf‐mtDNA fragmentomic signatures into a clinically applicable tool, we constructed an ED model using 170 features, which were selected via mutual information(MI) and Least Absolute Shrinkage and Selection Operator (LASSO) regression and integrated through partial least squares discriminant analysis (PLS‐DA) (Figure S14). In the training cohort, ED scores were significantly higher in EC patients than in the combined non‐EC groups (BED and HC; all *p* < 0.05; Figure S15A). The model yielded high discriminative power, with AUCs of 0.9833 for EC vs. HC and 0.9560 for EC vs. BED (Figure S15B). At an optimal cut‐off of 0.5167, which was determined by the Youden index, the model demonstrated a sensitivity of 90.18%, with specificities of 99.05% against HC and 89.13% against BED (Figure S15C,D). Given the high‐dimensional feature space, we performed nested cross‐validation to rigorously assess overfitting. The ED model yielded an unbiased outer‐loop AUC of 0.9473 (95% CI: 0.9314‐0.9631; Figure S15E), supporting model robustness against overfitting. Then the model's robustness was confirmed across multiple validation cohorts. In the internal validation cohort, ED scores remained significantly elevated in EC patients, with AUCs of 0.9944 (EC vs. HC), 0.9580 (EC vs. BED), and 0.9762 (EC vs. non‐EC), along with a specificity of 97.78% for HC and a sensitivity of 94.07% for EC, confirming the model stability without overfitting (Figure 3A–D; Table S4). In an independent external cohort, performance remained high, with AUCs of 0.9812, 0.9684, and 0.9748, respectively, and 93.33% sensitivity and 91.00% specificity against HC (Figure 3E–H). To assess robustness across genetic backgrounds, we tested the model in an ethnic minority cohort consisting predominantly of Uyghur and Kazakh individuals, which has distinct genetic and lifestyle profiles from the Chinese Han majority. The model retained strong performance, with AUCs of 0.9886, 0.9450, and 0.9668, alongside 90.00% sensitivity and 97.14% specificity against HC (Figure 3I–L) At the predefined cut‐off, sensitivity reached 90.00% with specificities above 88.00% across all cohorts. To assess clinical utility across diverse populations, we performed decision curve analysis (DCA) and precision‐recall (PR) curve analysis on combined internal, external, and minority validation cohorts. The ED model showed higher net benefit than “treat‐all” or “treat‐none” strategies across 0‐0.8 threshold probabilities (Figure S16A) and achieved an average precision (AP) of 0.9731 (Figure S16B). These results demonstrate that the ED model generalizes robustly across geographically and ethnically diverse populations, supporting its potential as a reliable diagnostic tool in varied clinical settings.

**FIGURE 3:**
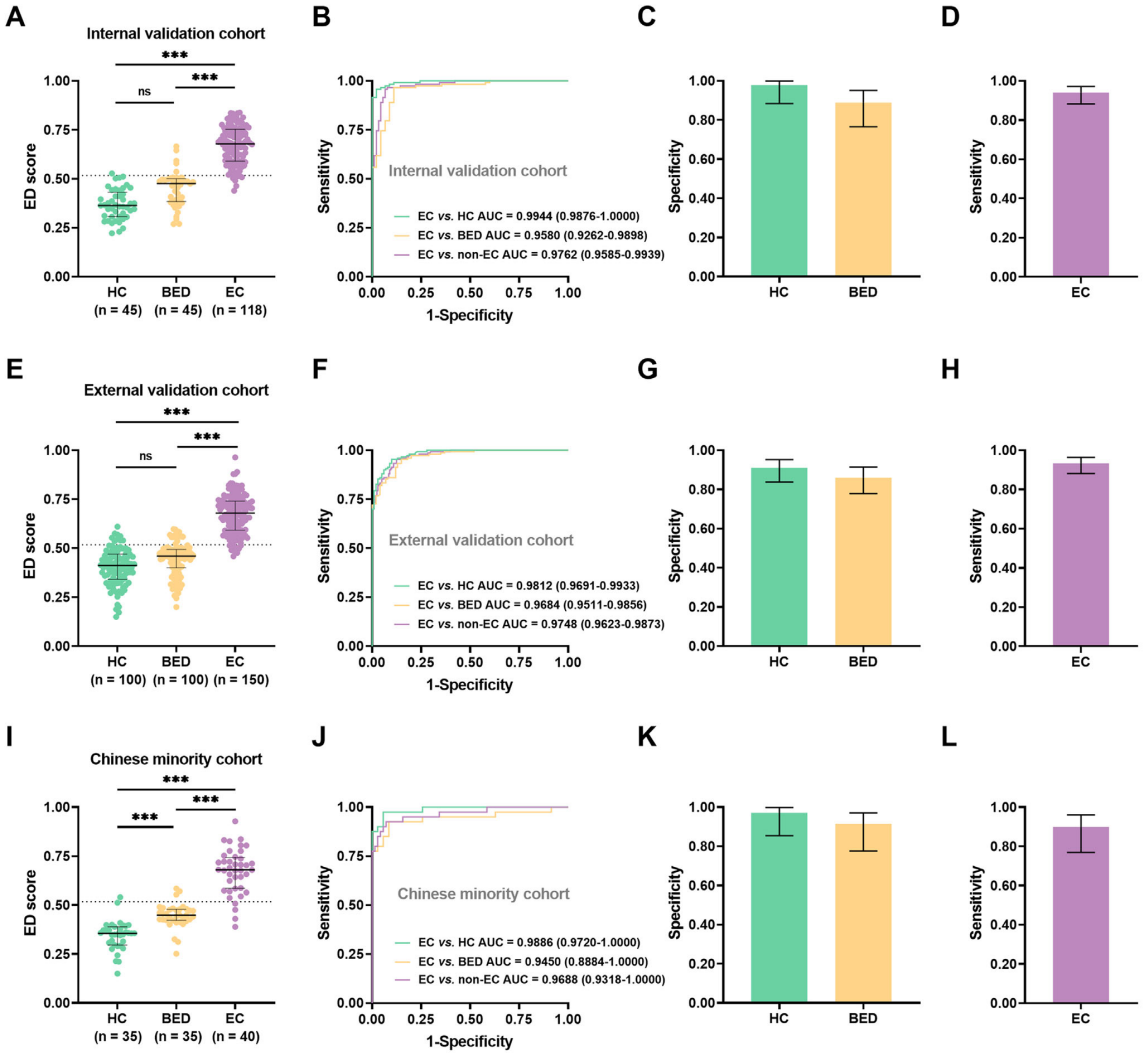
Performance of the ED model across three validation cohorts. ED score comparisons among the HC, BED and EC groups are presented across the (A) internal validation cohort, (E) external validation cohort and (I) Chinese minority cohort. The prespecified cut‐off (ED score = 0.5167) is indicated by the dashed line. Dots represent individual participants; horizontal lines indicate the median and IQR. (B, F, J) Receiver operating characteristic curves show performance in distinguishing EC from HC, BED, and non‐EC across three cohorts. (C, G, K) Specificity of the ED model in detecting HC and patients with BED. (D, H, L) Sensitivity of the ED model in detecting patients with EC. Error bars represent 95% CI. ns, not significant; ***, *p* < 0.001. HC, healthy control; BED, benign esophageal diseases; EC, esophageal cancer; ED, esophageal cancer detection; CI, confidence interval.

To further contextualize the performance of the ED model, we compared it with five representative blood‐based approaches previously reported for esophageal cancer detection, including cfDNA methylation, cfDNA fragmentomics, multi‐protein classifiers, metabolomics, and circulating microRNAs (Figure S17A) [20, 21, 22, 23, 24]. Although direct cross‐study comparisons should be interpreted cautiously due to differences in cohort composition and study design, the ED model achieved competitive performance in terms of AUC, sensitivity, and specificity (Figure S17B,C). These results support the potential clinical utility of ccf‐mtDNA fragmentomics as a complementary strategy for esophageal cancer detection.

To determine whether the ED model's high performance is specific to EC rather than reflecting a pan‐gastrointestinal cancer signal, we further evaluated the model on an independent cohort of patients with other gastrointestinal cancers, including 40 gastric cancer (GC) and 40 colorectal cancer (CRC) patients. At a cut‐off value of 0.5167, 95.00% of gastric cancer cases were correctly classified as true negatives, while 5.00% were false positives (misclassified as EC); 92.50% of CRC cases were correctly classified as true‐negatives, while 7.50% were false‐positives (Figure S18). These results demonstrate that the ccf‐mtDNA fragmentomic features captured by the ED model are specifically associated with esophageal cancer and do not simply represent a common malignancy‐associated signal.

### 3.4 The ED Model Maintains Consistent Performance Across Heterogeneous Clinical Factors

Having established cross‐center generalizability, we next assessed the ED model's performance across key clinical variables to ensure its robustness against the inherent heterogeneity of EC. ED scores remained stable across subgroups of HC, BED, and EC patients stratified by age, sex, smoking, alcohol use, BMI, or serum biomarkers (all *p* > 0.05; Figure S19), confirming the disease specificity of the fragmentomic features and their independence from common confounders. The model maintained high diagnostic accuracy across the spectrum of disease severity and presentation. ED scores increased progressively with disease advancement (*p* < 0.05). It achieved high accuracy for both early‐ and advanced‐stage disease, with AUCs of 0.9687 and 0.9817, and sensitivities of 92.90% and 93.53%, respectively (Figure 4A‐C; Table S5). Performance was similarly robust across tumor locations in the upper, middle, and lower esophagus, with AUCs of 0.9817, 0.9721, and 0.9751, respectively, and sensitivities above 92.00% (Figure 4D–F; Table S5). The model also demonstrated comparable accuracy for both major histological subtypes, with AUCs of 0.9752 for ESCC and 0.9688 for EAC, as well as corresponding high sensitivities of 92.75% and 96.88%, respectively (Figure 4G–I; Table S5). Beyond consistent subgroup performance, the model demonstrated significant clinical utility in addressing a key diagnostic challenge: the underestimation of severity due to biopsy sampling error. Among 29 patients whose preoperative biopsies showed only HGIN but whose final pathology confirmed early‐stage EC, the ED model correctly classified 93.10% (95% CI: 78.04–98.77%) as EC (Figure 4J,K; Table S5). This indicates that the model can detect invasive molecular features even when tissue sampling is limited or non‐representative, offering a valuable blood‐based adjunct to histopathology for guiding timely surgical intervention. Furthermore, the ED model achieved a sensitivity of 93.18%, which was significantly higher than the 21.1% sensitivity of conventional serum biomarkers (Figure S20). Collectively, these results affirm the ED model's robust and reliable performance across diverse, real‐world clinical scenarios, while its ability to reflect tumor burden and uncover occult invasion sets the stage for future studies on tracking disease progression from precancerous states.

**FIGURE 4:**
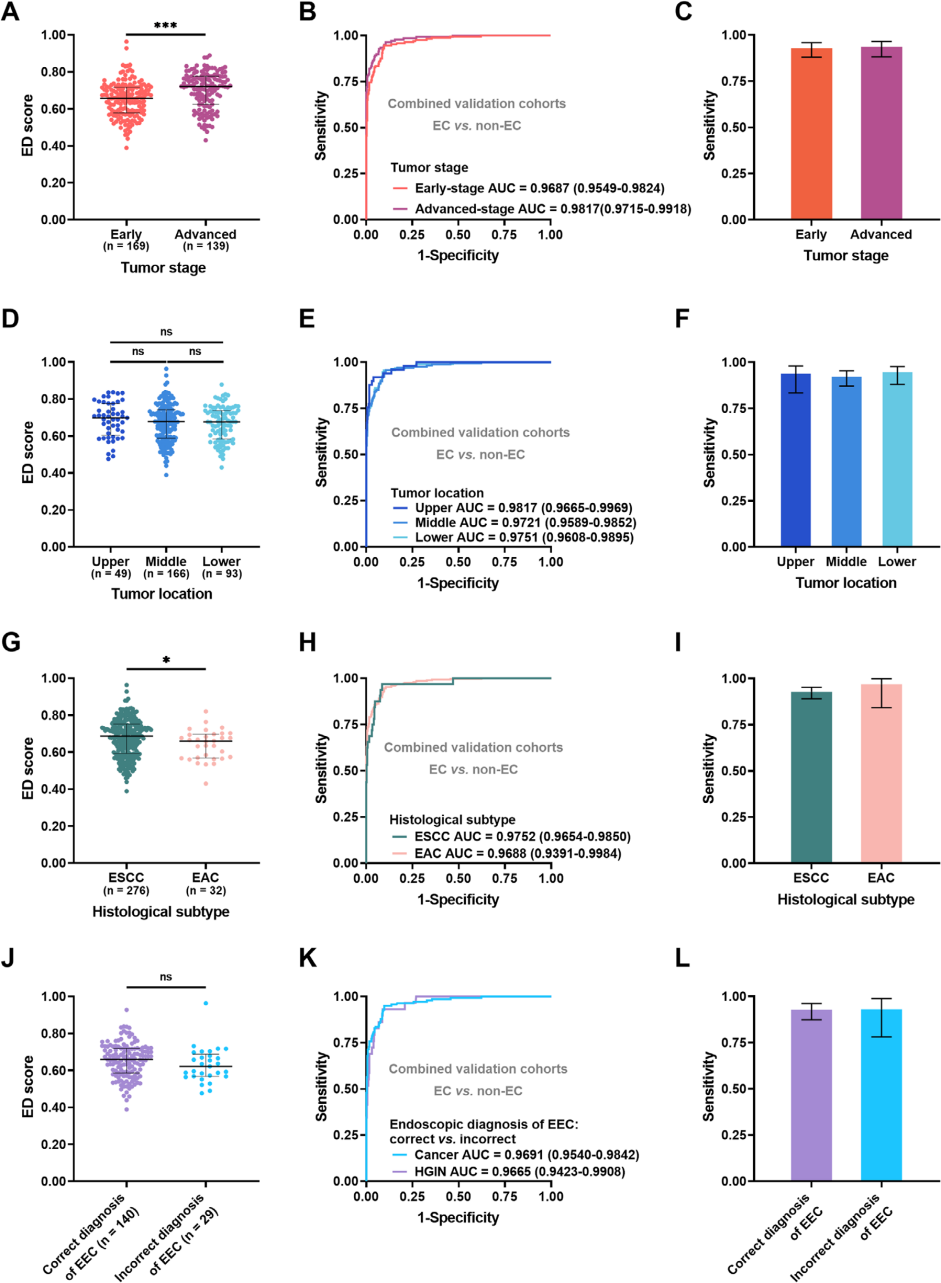
Performance of the ED model across clinical subgroups in the combined validation cohorts. The ED model was evaluated for discriminating EC from non‐EC (HC and BED) across clinical subgroups. ED score distributions are shown for (A) tumor stage (early‐stage and advanced‐stage), (D) tumor location (upper/middle/lower), (G) histological subtype (ESCC and EAC), and (J) endoscopic diagnosis of EEC (correct and incorrect). Dots represent individual participants; horizontal lines indicate the median and IQR. Receiver operating characteristic curves show performance in distinguishing EC from HC, EC from BED, and EC from non‐EC across (B) tumor stage, (E) tumor location, (H) histological subtype, and (K) endoscopic diagnosis, with the corresponding sensitivity of the ED model shown in (C), (F), (I), and (L), respectively. Error bars represent 95% CI. ns, not significant; *, *p* < 0.05; **, *p* < 0.01; ***, *p* < 0.001. HC, healthy control; BED, benign esophageal diseases; EC, esophageal cancer; EEC, early esophageal cancer; ED, esophageal cancer detection; ESCC, esophageal squamous cell carcinoma; EAC, esophageal adenocarcinoma; CI, confidence interval.

### 3.5 Plasma Ccf‐mtDNA Fragmentomics Reveals Distinct Molecular Profiles Across the Esophageal Neoplastic Spectrum

To examine whether ccf‐mtDNA fragmentomic patterns differ across stages of esophageal neoplasia, we performed a cross‑sectional analysis of plasma samples spanning (HC, BED, LGIN, HGIN, and EC). Uniform Manifold Approximation and Projection (UMAP) visualization separated non‑EC from EC samples, with HGIN cases located within an intermediate transitional zone (Figure 5A). Quantitative changes in regional fragmentomic profiles further supported a stepwise shift along the disease continuum (Figure 5B). Consistently, Jonckheere‑Terpstra trend tests identified multiple fragmentomic features that changed monotonically across the spectrum from non‑EC to LGIN, HGIN, and EC (all *p* < 0.05; Figure 5C), indicating gradual molecular progression preceding histological malignancy. The ED model effectively captured this progression, achieving 90.0% accuracy in classifying LGIN and HGIN (Figure 5D). ED scores exhibited a significant stepwise increase correlating with pathological severity, rising from 0.40 in HC to 0.68 in EC patients (*p* < 0.05; Figure 5E). Furthermore, the ED model discriminated HGIN and LGIN from the non‐EC group with AUCs of 0.9597 and 0.5917, respectively (Figure 5F, Table S6). Discordant classifications were often associated with lesion size, suggesting that fragmentomic features reflect global molecular burden beyond focal biopsy sampling. Clinically, decision‑curve analysis across threshold probabilities of 0.1‐0.8 indicated that ED model‑guided triage provided greater net benefit than universal biopsy or no‑biopsy strategies (Figure 5G), supporting its potential for risk‑stratified screening. Together, these results reveal invasion associated ccf‑mtDNA fragmentomic signatures across the esophageal neoplastic continuum and demonstrate the capacity of the ED model to detect precancerous lesions.

**FIGURE 5:**
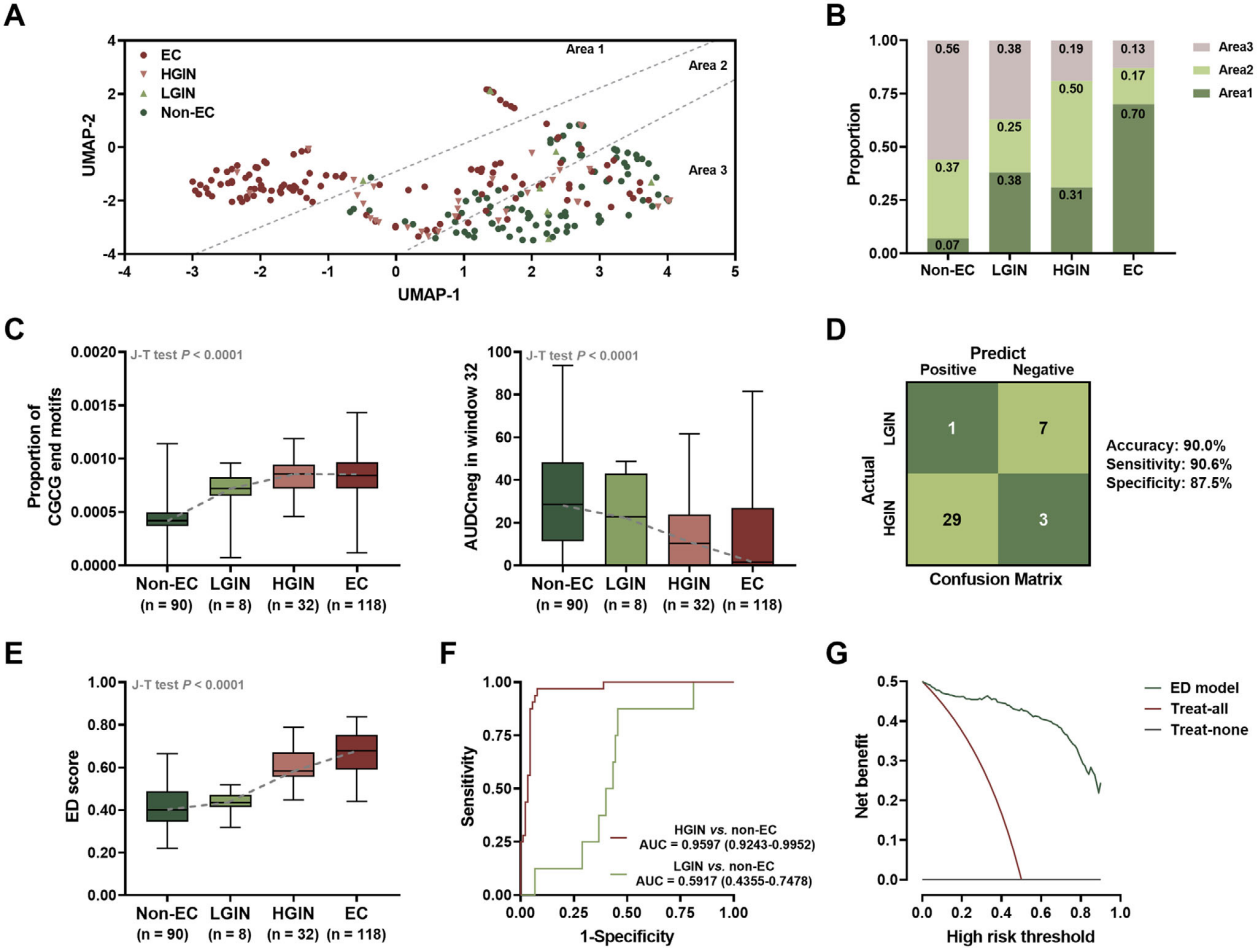
Evaluation of the ED model for detecting precancerous lesions. (A) Uniform manifold approximation and projection of samples based on ED‐model features showing separation among non‐EC (HC and BED), LGIN, HGIN, and EC groups. Dashed lines delineate three predefined regions (Areas 1–3). (B) Proportional distribution of samples from each pathological group across Areas 1–3. (C) The proportion of CGCG‐end motifs exhibited a significant stepwise elevation along with disease progression, while AUDCneg in window 32 displayed a concomitant decline (Jonckheere‐Terpstra test, *p* < 0.0001). (D) Confusion matrix summarizing classification results of the ED model; numbers indicate correctly and incorrectly classified LGIN and HGIN samples. (E) Distributions of ED score across four pathological stages. (F) Receiver operating characteristic curve showing the performance of the ED model for discriminating LGIN and HGIN from non‐EC. (G) Decision curve analysis showing net clinical benefit of the ED model across a range of high risk thresholds, compared with biopsy‐all and biopsy‐none strategies. HC, healthy control; BED, benign esophageal diseases; EC, esophageal cancer; ED, esophageal cancer detection; LGIN, low‐grade intraepithelial neoplasia; HGIN, high‐grade intraepithelial neoplasia; AUDCneg, Area Under the Distribution Curve of the negative peaks.

### 3.6 Plasma Ccf‐mtDNA Fragmentomics‐Based Model Enables Non‐Invasive Discrimination Between ESCC and EAC

Given the divergent treatment strategies for ESCC and EAC, accurately distinguishing between these subtypes is critical for clinical decision‐making. To address this need without relying solely on invasive biopsy, we investigated whether ccf‐mtDNA fragmentomics could enable non‐invasive molecular subtyping. Initial screening of 1,365 fragmentomic features in 583 patients (527 ESCC, 56 EAC) revealed subtype‐specific molecular landscapes, with 9 features elevated and 27 decreased in ESCC relative to EAC (Figure 6A). To address the marked class imbalance between ESCC and EAC, we combined oversampling of the minority EAC class with cost‐sensitive learning during model fitting, followed by a two‐step feature‐selection procedure consisting of MI filtering and LASSO regression (Figure S21). We further compared LASSO models using the lambda.1se and lambda.min criteria to assess the robustness of feature selection. The more conservative lambda.1se criterion reduced the number of retained features from 336 to 126, while maintaining comparable performance in both the development and independent validation cohorts (Figure S22). As lambda.1se did not improve validation performance, lambda.min was retained for the primary PSP model. Using the selected features, we constructed a PSP model based on logistic regression with cost‐sensitive learning to mitigate bias against the minority class (EAC). Under 5‐fold cross‐validation, the PSP model achieved an AUC of 0.9522 and accuracy of 89.71% (Figure 6B,C and Table S7). To verify generalization independent of cross‐validation splits, we performed 5×5 nested cross‐validation for the PSP model in the combined ESCC/EAC cohort. The unbiased outer‐loop AUC was 0.9504 (95% CI: 0.8776‐1.0000; Figure 6D), supporting the robustness of model generalization. DCA confirmed that the PSP model provided higher net clinical benefit than a uniform ESCC classification strategy across probability thresholds of 0.1‐0.8 (Figure 6E). The PR curve yielded AP of 0.9945 (Figure 6F), indicating excellent reliability for distinguishing ESCC from EAC even with the minority EAC class. The PSP model was further evaluated in the independent validation cohort using the prespecified decision threshold of 0.7603, without any model refitting or threshold re‐optimization. At this predefined threshold, the model achieved a sensitivity of 87.50% (95% CI: 73.89–94.54%) for ESCC and a specificity of 90.00% (95% CI: 76.95–96.04%) for EAC (Table S7). The model also demonstrated strong overall discrimination, with an AUC of 0.9231 (95% CI: 0.8557‐0.9905; Figure 6G), an overall accuracy of 88.75% (Figure 6H), and an AP of 0.9432 (Figure 6I). These results demonstrate that the predefined PSP model and decision threshold retain robust performance in previously unseen patients, supporting the potential of ccf‐mtDNA fragmentomics for non‐invasive EC subtyping.

**FIGURE 6:**
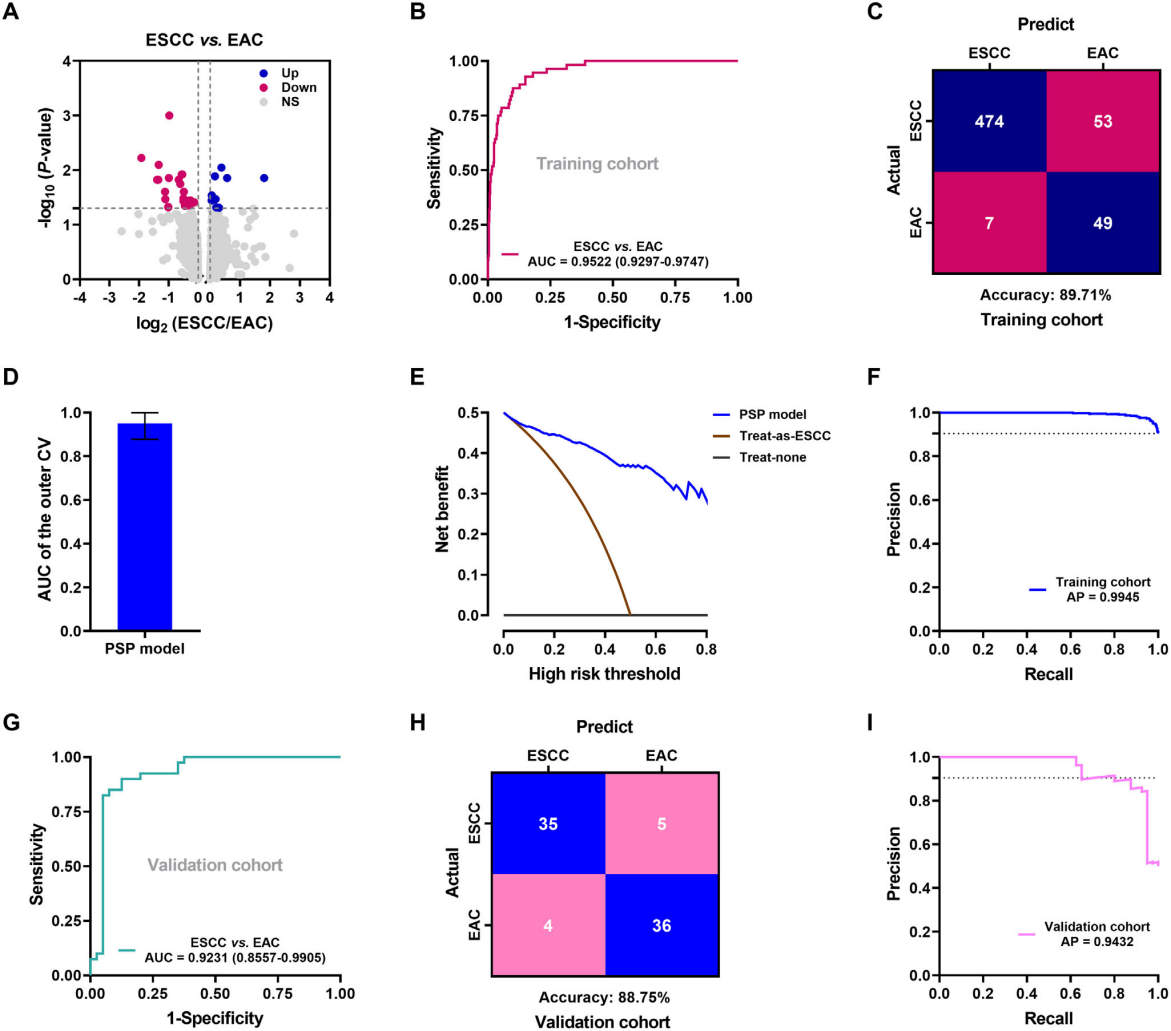
Performance of the PSP model for predicting pathological subtype. (A) Volcano plot of differentially abundant fragmentomic features between ESCC and EAC. Each point represents one feature; dashed lines indicate the thresholds used to define differentially abundant features; a positive log_2_ fold change indicates enrichment in ESCC. (B) ROC curve showing the performance of the PSP model in distinguishing ESCC from EAC. (C) Confusion matrix summarizing classification results of the PSP model; numbers indicate correctly and incorrectly classified ESCC and EAC samples. (D) AUC values on the outer CV test set, error bars represent 95% CI. (E) Decision curve analysis showing the net clinical benefit of the PSP model across a range of high risk thresholds, compared with the treat‐as‐ESCC and treat‐none strategies. (F) PR curves of the PSP models, with AP values. (G) ROC curve of the PSP model in the validation cohort. (H) Confusion matrix summarizing classification results of the PSP model on the validation cohort; numbers indicate correctly and incorrectly classified ESCC and EAC samples. (I) PR curves of the PSP models on the validation cohort, with AP values. PSP, pathological subtype prediction; ESCC, esophageal squamous cell carcinoma; EAC, esophageal adenocarcinoma; ROC, receiver operating characteristic; AUC, area under the curve; PR, precision‐recall; AP, average precision; CV, cross‐validation.

To further enhance precision, we introduced a dual‑threshold “grey zone” to identify cases with uncertain PSP scores. For these, a rescue model was developed that integrated the original PSP score with key clinical parameters, including BMI, smoking, alcohol history, gastroesophageal reflux disease (GERD) history and serum biomarkers (SCCA, CEA, carbohydrate antigen 19‐9, cytokeratin fragment antigen 21‐1). The resulting modified PSP (mPSP) score maintained reliable PSP predictions outside the grey zone while rescuing uncertain cases. This integrated approach improved performance, with the mPSP model achieving an AUC of 0.9570 and accuracy of 90.74% (Figure S23A,B and Table S7). Multi‐variable analysis confirmed that the PSP score remained an independent predictor of subtype after adjusting for clinical variables (Wald = 12.23, *p* < 0.05, Table S8). Similarly, nested cross‐validation (5×5 design) was performed for the mPSP model. The unbiased outer‐loop AUC was 0.9435 (95% CI: 0.8924‐0.9945; Figure S23C), confirming that integrating clinical variables does not introduce overfitting. We further performed DCA and PR curve analysis for the mPSP model on the multi‐center validation cohorts. DCA demonstrated superior net benefit compared to default strategies across a wide range of threshold probabilities (Figure S23D). The PR curve achieved an AP of 0.9951 (Figure S23E). To further assess the robustness and generalizability of the mPSP model, we evaluated the fully locked model in the independent validation cohort using its prespecified decision threshold of 0.4910, without model refitting or threshold re‐optimization. At this threshold, the mPSP model achieved a sensitivity of 95.00% (95% CI: 83.50–99.11%) for ESCC and a specificity of 90.00% (95% CI: 76.95–96.04%) for EAC (Table S7). The model achieved an AUC of 0.9406 (95% CI: 0.8767‐1.0000; Figure S23F) and an overall accuracy of 92.50% (Figure S23G), while PR‐curve analysis yielded an AP of 0.9605 (Figure S23H). These results further support the robustness of the mPSP model for independent pathological subtype prediction. Subgroup analyses demonstrated that the mPSP model exhibited robust predictive performance (Figure S24). These results demonstrate that ccf‑mtDNA fragmentomics, especially when integrated with clinically accessible variables, can effectively and non‑invasively discriminate ESCC from EAC, offering a valuable tool for guiding subtype‑specific management of EC.

## 4 Discussion

EC remains a significant global health challenge, largely due to the lack of reliable non‑invasive methods for early detection and precise subtyping [25, 26]. In this large‑scale, multi‑center study, we developed and validated a diagnostic framework based on plasma ccf‑mtDNA fragmentomics. This framework successfully addresses two critical unmet needs in EC management: (1) the non‑invasive identification of HGIN and early‑stage carcinoma, and (2) the accurate discrimination between ESCC and EAC to guide subtype‑specific therapy. Our results establish ccf‑mtDNA fragmentomics as a robust biomarker that captures the molecular continuum of esophageal carcinogenesis.

Compared with previously reported cfDNA‐based screening approaches, the present strategy possesses several distinctive technical characteristics. Unlike methylation‐based assays, ccf‐mtDNA fragmentomic analysis does not require bisulfite conversion and therefore preserves native DNA fragmentation information. In addition, targeted mitochondrial capture enables substantially deeper mtDNA coverage than conventional WGS‐based approaches, facilitating high‐resolution characterization of ccf‐mtDNA fragmentation patterns. Given the naturally high copy number of mitochondrial DNA in circulation, this approach may provide robust molecular signals even in early‐stage disease with limited ctDNA shedding.

The exceptional cross‐population robustness of the ED model constitutes a major translational strength of our study. While biomarker generalizability is often limited by genetic and environmental heterogeneity [27], our model maintained consistently high performance (AUCs: 0.9450‐0.9944) across rigorously validated cohorts from Eastern, Northwestern, and multi‐ethnic regions of China, including a dedicated cohort of Uyghur and Kazakh individuals from Xinjiang. This cross‐population stability supports the reproducibility of the underlying fragmentomic signatures and suggests that they may reflect conserved biological processes associated with esophageal tumorigenesis, including mitochondrial dysfunction and metabolic reprogramming, rather than population‐specific artifacts.

Beyond binary detection, our fragmentomic analysis provides a molecular view of stepwise esophageal carcinogenesis. The fragmentomic landscape revealed a clear continuum, with HGIN occupying an intermediate “transitional zone” between benign tissue and invasive cancer. This aligns with the concept of field cancerization and suggests that ccf‑mtDNA fragmentation patterns progressively change across the esophageal neoplastic continuum. The ED model's high sensitivity for HGIN of 90.63% and its ability to resolve biopsy‑discordant cases underscore its clinical utility. By reflecting the total molecular burden and transcending the constraints of focal sampling, this blood‐based strategy identifies high‐risk patients for early resection, thereby optimizing clinical resources in high‐prevalence areas.

The biological basis underlying these disease‐associated ccf‐mtDNA fragmentation alterations may involve dysregulated DNA processing. Previous studies have shown that cfDNA fragmentation, including fragment size and end‐motif patterns, is influenced by nuclease‐mediated cleavage, with distinct nucleases generating characteristic fragment‐end signatures [28, 29, 30], and DNASE1, DNASE1L3, DFFB, and ENDOG have established roles in DNA processing [28, 29, 31, 32, 33]. In line with this biological framework, our finding of disease‐associated alterations in circulating nuclease profiles provides additional support for a potential contribution of nuclease dysregulation to the aberrant ccf‐mtDNA fragmentation observed across the esophageal disease spectrum.

Remarkably, accurate histological subtyping (distinguishing ESCC from EAC) is critical for personalized treatment [5]. Here, we demonstrate for the first time that ccf‐mtDNA fragmentomics encodes sufficient information to differentiate these subtypes with high accuracy (PSP model: AUC = 0.9522). Importantly, the prespecified PSP and mPSP decision thresholds maintained high sensitivity and specificity in an independent multicenter validation cohort, providing direct evidence that their operating characteristics generalize beyond the development dataset. ESCC and EAC arise from distinct cellular origins and exhibit divergent metabolic and mitochondrial states. ESCC is associated with increased oxidative stress and frequent TP53 alterations, which may enhance mitochondrial DNA damage and influence fragmentation profiles [7, 14, 34]. In contrast, EAC commonly develops from Barrett's esophagus, a metaplastic condition associated with mitochondrial dysfunction and altered expression of mtDNA‐binding proteins such as TFAM [14, 34]. Notably, plasma concentrations of DNASE1L3, DNASE1, and DFFB also differed significantly between EAC and ESCC. Against the broader background of nuclease dysregulation observed across the esophageal disease spectrum, these subtype‐associated differences suggest that distinct DNA‐processing states may further contribute to the divergent ccf‐mtDNA fragmentation patterns of ESCC and EAC. Acknowledging the complexity of real‐world clinical decision‐making [35], we further integrated these molecular features with routine clinical parameters (e.g., BMI, GERD history, serum biomarkers) to create a refined mPSP model. In patients with suspected or confirmed EC, the PSP model may provide complementary non‐invasive information for pathological subtyping and clinical referral. For patients with inconclusive pathological findings due to insufficient biopsy material, or those unable to tolerate invasive examinations, the mPSP model integrating clinical information may further support clinical decision‐making. This framework may facilitate more personalized management of EC and warrants further prospective evaluation for clinical translation.

Based on these findings, we propose a tiered clinical workflow. In primary care or high‑risk population screening, the ED model could act as an efficient triage tool, guiding the need for endoscopy and reducing unnecessary procedures. In patients with ambiguous symptoms or imaging findings, the ED score can serve as a molecular severity index. For patients with confirmed EC where histologic subtyping is challenging, the mPSP model offers a reliable, non‑invasive alternative to inform treatment selection, including neoadjuvant therapy strategies. The high sensitivity of the ED model may be particularly valuable for risk stratification in settings where endoscopic resources are limited.

In our study, four limitations should be considered. First, the retrospective design requires prospective validation of the model's ability to predict progression from LGIN to invasive cancer. Second, although the newly added circulating nuclease protein measurements provide an additional biological layer supporting systemic nuclease dysregulation in EC, paired multi‐omics profiling from the same individuals and direct in vitro or in vivo causal evidence linking individual nucleases to specific ccf‐mtDNA fragmentation patterns remain unavailable. Therefore, the observed associations should not be interpreted as establishing causality. Future studies integrating nuclease activity assays, paired molecular profiling, and functional perturbation experiments will be required to define the underlying mechanisms. Third, external validation in global populations is needed to strengthen generalizability beyond the ethnically diverse cohorts within China studied here. Finally, due to the cross‐sectional design of the current study, longitudinal follow‐up data are not yet available. Therefore, the predictive value of the ED and PSP/mPSP scores for disease progression, recurrence, and prognosis (e.g., progression‐free survival, overall survival) cannot be assessed at this stage. We will initiate a prospective longitudinal cohort and evaluate these endpoints in future studies.

Beyond the limitations discussed above, several mechanistic questions raised by our findings warrant further investigation. First, the cellular origin of ccf‐mtDNA, whether derived from tumor cells or from inflammation/stress‐induced release by non‐malignant cells, cannot be definitively determined at the current bulk‐sequencing resolution. Future studies will need to develop approaches that achieve single‐molecule resolution or leverage cell‐type‐specific molecular patterns to distinguish the origin of individual mtDNA molecules. Second, the molecular basis underlying EC‐specific fragmentomic signatures, such as elevated TGTT/TGT terminal motifs, remains to be elucidated. Previous studies have shown that cfDNA fragmentation is strongly influenced by nuclease‐mediated cleavage [34, 35], and our previous work demonstrated altered levels of several key nucleases, including deoxyribonuclease 1 (DNASE1), DNASE1L3, DNA fragmentation factor subunit beta (DFFB), and ENDOG, in patients with neoplastic diseases [19]. Together with the circulating nuclease alterations observed in the present study, these findings raise the possibility that altered nuclease abundance may contribute to aberrant ccf‐mtDNA fragmentation in EC. However, direct causal relationships remain to be established through functional experiments, nuclease activity assays, and multi‐omics integration. Notably, although global fragmentomic profiles did not significantly differ between early‐ and advanced‐stage EC, 124 specific features exhibited stage‐dependent changes. One possible explanation is that mitochondrial dysfunction may occur early during esophageal carcinogenesis and contribute to stable alterations in ccf‐mtDNA fragmentomic profiles. However, this hypothesis requires direct experimental validation.

Collectively, our findings support ccf‐mtDNA fragmentomics as a promising complementary liquid‐biopsy strategy for esophageal cancer detection and pathological subtyping. By integrating disease‐associated fragmentomic patterns with clinically accessible information, this approach provides a foundation for future prospective studies aimed at refining non‐invasive risk stratification and personalized management of EC.

## Author Contributions

Yang L. and J.X. conceived and designed the study. Yue L., W.Y., Y.L., S.W., F.P., M.D., H.Z., H.J., Y.Y., Z.S., X.M., and R.L. recruited and enrolled participants, collected samples and clinical data, and performed mtDNA sequencing. Yue L., W.Y., L.Y., S.W. and H.J. analyzed and interpreted the sequencing data. Yue L. and W.Y. drafted the manuscript. Yang L. and J.X. supervised the study and revised the manuscript. Yang L., J.X., Xiang L. and Yue L critically revised the manuscript. All authors reviewed and approved the final manuscript. Yang L. and J.X. are the corresponding authors.

## Funding

This work was supported by the National Natural Science Foundation of China (grant no. 82330073, 82402731), the Science & Technology Co‐ordination and Innovation Project of Shaanxi Province, China (grant no. 2024SF‐ZDCYL‐02‐02), Clinical Research Project of Air Force Medical University (2023LC2324 and 2023LC2315), Noncommunicable Chronic Diseases‐National Science and Technology Major Project (2023ZD0510900). National Science and Technology Major Project Youth Talent Cultivation Program (No. SQ2023AAA031957).

## Ethics Statement

This study was approved by the Institutional Review Board of Tangdu Hospital of Fourth Military Medical University (permission number: KY20183331‐1; Date issued: 2018‐03‐08), Fudan University Shanghai Cancer Center (permission number: 050432‐4‐2108; Date issued: 2021‐08‐09) and Tumor Hospital Affiliated to Xinjiang Medical University (permission number: S‐2024113; Date issued: 2024‐07‐24). Written informed consent was obtained from all participants.

## Conflicts of Interest

The authors declare no conflicts of interest.

## Supporting information




**Supporting File 1**: advs78377‐sup‐0001‐SuppMat.docx.

**Supporting File 2**: advs78377‐sup‐0002‐SuppMat.docx.

**Supporting File 3**: advs78377‐sup‐0003‐SuppMat.docx.

## Data Availability

The raw sequencing data underlying this article are available in BIG Data Center, Beijing Institute of Genomics (BIG) with access number PRJCA055903. All code used for the analyses and visualizations in the manuscript is available at https://github.com/Mitoomics‐EC/code.
