# Supplementary material for: Noninvasive Detection and Subtyping of Esophageal Cancer by Circulating Cell‐Free mtDNA Fragmentomics: A Multi‐Center Validation Study
Source: Adv Sci (Weinh). 2026 Oct 28:e78377. Online ahead of print. doi: 10.1002/advs.78377 (PMC13715668; doi:10.1002/advs.78377)
Supplement: Supplementary file 1 [file ADVS-9999-e78377-s001.docx]

**Methods**

**Participant enrollment and study design**

Between May 2023 and September 2026, a total of 1,260 participants were enrolled in this multi-center study from three hospitals in China: Tangdu Hospital of the Fourth Military Medical University (FMMU), Fudan University Shanghai Cancer Center, and Tumor Hospital Affiliated to Xinjiang Medical University. The cohort consisted of patients with esophageal cancer (EC), esophagus intraepithelial neoplasia, or benign esophageal diseases (BED), as well as healthy controls (HC).

The inclusion criteria were as follows: (1) age between 18 and 85 years; (2) patients with EC had pathologically confirmed diagnoses according to the World Health Organization (WHO) classification; (3) BED patients, including those with esophagitis, gastroesophageal reflux disease, and Barrett’s esophagus without dysplasia, were diagnosed based on clinical evaluation and confirmatory endoscopic and histopathological findings consistent with ASGE/ESGE guidelines and had no history of malignancy; (4) Patients had pathologically confirmed high-grade or low-grade intraepithelial neoplasia; (5) HC were recruited to match the age and sex distribution of patients with EC, with no history of malignancy or clinical symptoms suggestive of esophageal disease; (6) all participants provided peripheral blood samples, completed a structured questionnaire on lifestyle and relevant clinical information, and provided written informed consent. The exclusion criteria were as follows: (1) presence of concomitant malignancies in other organ systems, significant chronic systemic diseases, or severe infectious diseases; (2) pregnancy, or lactation.

Tumors were staged according to the 8th edition of the American Joint Committee on Cancer (AJCC) TNM staging system for EC. Early-stage EC (EEC) was defined as stage I or II disease, whereas advanced-stage esophageal cancer (AEC) was defined as stage III or IV. The study protocol was approved by the ethics committees of all participating centers. Detailed demographic and pathological characteristics of the study population are summarized in Supplementary Table 1.

**Sample collection, DNA extraction, library construction and sequencing**

Peripheral blood samples (5 mL) were collected from each participant into K2-EDTA tubes before endoscopy or any treatment initiation. Plasma was separated within 2 hours using a standard two-step centrifugation protocol and stored at −80℃ until further processing. All three research centers adopted a unified standard operating procedure for sample handling. In addition, inter‑center sample transport was carried out on dry ice to preserve sample integrity. Samples from different centers and disease groups were randomly pooled and processed together. Circulating cell-free DNA (cfDNA) was extracted from plasma using the VAMNE MagUltra Circulating Cell-free DNA Isolation Kit (Vazyme, China) and quantified using a Qubit 4.0 (Thermo Fisher, USA). Sequencing libraries were constructed from 10-20 ng of cfDNA and subjected to hybrid capture with custom-designed biotinylated mtDNA probes, following established protocols^[1]^. The ccf-mtDNA libraries were sequenced on an Illumina NovaSeq 6000 platform (Illumina, USA) with paired-end 150 bp reads. Library preparation was performed in 13 batches, each containing a balanced mix of EC and non-EC samples to minimize potential batch effects.

**Analysis of ccf-mtDNA fragmentomic features**

Raw sequencing data processing and alignment followed our previously established pipeline^[1, 2]^. Briefly, low-quality bases and adapters were trimmed using fastp (v.1.0.1). We set a window average quality threshold of Q30 and an overall read average quality threshold of Q30, and retained high-quality reads with a post-trimming length of ≥50 bp. To minimize nuclear mitochondrial DNA sequences (NUMTs) contamination, reads were simultaneously aligned to the revised Cambridge Reference Sequence (rCRS) and the human reference genome (hg19) using BWA (v0.7.12) in MEM mode with default parameters, retaining only reads uniquely mapped to rCRS. Picard Tools software (v.1.119) was used to sort the reads, and duplicate reads were removed using Picard Mark Duplicates software (v.1.81). Notably, no dedicated filtering was applied to reads with TLEN=0 due to their extremely low abundance, especially after alignment to the personalized reference genome. The average mtDNA coverage depth of all samples was 3,923× (Supplementary Table 3). Fragments covering positions 1-16569 of the mitochondrial genome were categorized into short or long groups based on the sample-specific median values as previously described^[1, 2]^. The fragment size distribution (FSD) score, computed as the ratio of long-to-short fragment coverage depths and adjusted for GC content via LOESS regression (span=0.75, degree=1), was normalized using z-score transformation to generate a fragmentation profile across all positions. The HC baseline was established using the median fragmentation profile from a randomly selected cohort of 30 healthy controls. Then, ccf-mtDNA fragmentomic features were extracted according to our previously published framework, including:

(i) Correlation with the HC median profile: Pearson correlation between the sample’s fragmentation profile and HCs baseline profile;
(ii) New peaks: Number of peaks present in the sample but absent in the median HC profile;
(iii) Total peaks: Total number of peaks (local maxima with width >5 bp) in the fragment size distribution (FSD) score curve;

(iv) Similar peaks: similar number of peaks (local maxima with width >5 bp) in the fragment size distribution (FSD) score curve;

(v) Euclidean Distance per window: To map local deviations, the mitochondrial genome was divided into 255 windows. For each window, the Euclidean Distance between the sample’s normalized coverage vector and the median HC vector was computed;

(vi) Area Under the Distribution Curve of negative and positive peaks per window: The mitochondrial genome was equally divided into 255 windows. The areas of the positive and negative peaks were calculated for each window at a baseline FSD score of 0;
(vii) Regional Pattern Consistency per window: To quantify local topological coherence, the mitochondrial genome was divided into 255 windows. For each window, the Pearson correlation coefficient between the sample's FSD vector and the median HC reference vector was computed;

(viii) Standardized end‑base proportions: (observed proportion of fragments ending with a specific base) / (genomic frequency of that base in the mtDNA reference). Values >1 indicate cleavage preference;
(ix) 5′‑end motif proportions: Frequency distributions of 2‑mer, 3‑mer, and 4‑mer motifs at fragment 5′ ends, reflecting longer sequence‑context preferences;

(x) 5′end Motif Diversity Score (MDS): Score (0-1) reflecting sequence‑specific cleavage preference at fragment 5′ ends based on 4‑mer terminal motif distribution. 0 = extreme bias, 1 = no bias.

**Measurement of concentrations of four nucleases in plasma**

The plasma concentrations of deoxyribonuclease 1 (DNASE1), deoxyribonuclease 1-like 3 (DNASE1L3), DNA fragmentation factor subunit beta (DFFB), and endonuclease G (ENDOG) were measured in plasma samples from 15 healthy controls, 15 patients with BED, 15 patients with ESCC, and 15 patients with EAC using commercially available enzyme-linked immunosorbent assay (ELISA) kits (Chang Lunshuo, China) according to the manufacturer’s instructions. The lower detection limits were 0.1562 ng/mL for DNASE1, 0.5 ng/mL for DNASE1L3, 0.0312 ng/mL for DFFB, and 0.6250 ng/mL for ENDOG. The assay plates were read at 450 nm. Each plasma sample was measured in triplicate, and the mean of the three measurements was used for statistical analysis.

**Construction and validation of the EC detection model**

Participants from Tangdu Hospital were enrolled and then randomly partitioned into training and internal validation cohorts at a 7:3 ratio. In the training cohort, we employed a two-step feature selection strategy for dimensionality reduction. First, a total of 1,365 features were ranked based on their mutual information (MI) with the outcome, and the top 50% were retained. Subsequently, the Least Absolute Shrinkage and Selection Operator (LASSO) regression was applied to this subset. The optimal regularization parameter (λ) was determined via cross-validation, yielding a final set of 170 non-zero coefficients as the selected features for distinguishing between EC and non-EC. Using the selected features, a discriminant model, which was designated as the EC detection (ED) model was constructed using partial least squares discriminant analysis (PLS-DA). The resulting scores were used as a continuous metric for EC detection. The performance of ED model was evaluated using ten-fold cross-validation in the training cohort. The optimal diagnostic threshold for distinguishing EC from non-EC individuals was determined based on the maximum Youden index in the training cohort. Based on the threshold, the performance of the ED model, including sensitivity and specificity was evaluated systematically in the internal validation cohort. The model's robustness and generalizability were further validated in two independent cohorts: (i) an external validation cohort comprising 350 participants recruited from Fudan University Shanghai Cancer Center; and (ii) a Chinese minority cohort comprising 110 participants recruited from Tumor Hospital Affiliated to Xinjiang Medical University. No information from the internal, external, or ethnic minority validation cohorts was used during feature selection or model training.

In addition, to assess the specificity of the ED model for EC, we included a control cohort of 40 patients with gastric cancer (GC) and 40 patients with colorectal cancer (CRC). These patients were randomly selected from our previously published studies ^[1,3]^, which used the identical capture-based ccf-mtDNA sequencing protocol as the current study. All GC and CRC patients had pathologically confirmed diagnoses without concurrent esophageal malignancy. Detailed demographic and pathological characteristics of the study population are summarized in Supplementary Table 2.

**Performance evaluation of the ED model in precancerous cohort**

To validate the performance of the ED model in identifying precancerous lesions, 8 patients with LGIN and 32 patients with HGIN were enrolled in a precancerous validation cohort from Tangdu Hospital. The ED scores were calculated using the ED model. Then, the Receiver Operating Characteristic (ROC) curve was analyzed for distinguishing HGIN from the HC and BED group.

**Construction and validation of the pathological subtype prediction model**

To enable non-invasive discrimination between ESCC and EAC, we developed a pathological subtype prediction (PSP) model using ccf-mtDNA fragmentomic features from the combined development cohort, comprising 527 patients with ESCC and 56 patients with EAC. To address the marked class imbalance between ESCC and EAC, we applied two complementary strategies during model development. First, the minority EAC class was oversampled within the training data to reduce class imbalance. Second, cost-sensitive learning was incorporated into the logistic regression model, with class-balancing weights derived from the relative sample sizes and additional penalties assigned to misclassification of the minority EAC class.

Feature selection was performed in two sequential steps. First, all 1,365 fragmentomic features were ranked according to their mutual information (MI) with pathological subtype, and the top 80% were retained. Second, least absolute shrinkage and selection operator (LASSO) regression was applied to the retained feature set. The regularization parameter (λ) was selected by cross-validation. For the primary analysis, the lambda.min criterion was used, yielding 336 features with non-zero coefficients. To assess the robustness of feature selection and the potential impact of model complexity, we additionally performed a sensitivity analysis using the more conservative one-standard-error (lambda.1se) criterion, which reduced the number of retained features to 126. PSP models were then constructed separately using the features selected under the lambda.min and lambda.1se criteria, and their predictive performance was compared in both the development and independent validation cohorts. Because the lambda.1se-based model did not improve validation performance relative to the lambda.min-based model, the lambda.min feature set was retained for the primary PSP model. The selected fragmentomic features were entered into a logistic regression model to generate the PSP score, and model performance was initially assessed using five-fold cross-validation.

To improve classification of cases with uncertain PSP scores, we further developed a modified PSP (mPSP) strategy based on a dual-threshold “grey zone.” Patients with PSP scores falling within this grey zone were re-evaluated using a multivariable logistic regression rescue model that incorporated the original PSP score together with clinically accessible variables, including body mass index (BMI), smoking history, alcohol consumption, gastroesophageal reflux disease (GERD) history, and serum tumor markers, including squamous cell carcinoma antigen (SCCA), carcinoembryonic antigen (CEA), carbohydrate antigen 19-9 (CA19-9), and cytokeratin 19 fragment (CYFRA21-1). For patients with PSP scores outside the grey zone, the original PSP score was retained as the final mPSP score. The performance of the mPSP strategy was assessed using five-fold cross-validation.

Decision thresholds for the PSP and mPSP models were determined exclusively in the development cohort before evaluation of the independent validation cohort. Based on prespecified clinical utility criteria, we required a minimum sensitivity of approximately 89% for ESCC and then selected the threshold that maximized specificity for EAC. After threshold determination, the selected features, model parameters, and decision thresholds were fully locked, and no feature reselection, model refitting, hyperparameter tuning, or threshold re-optimization was performed using the independent validation data.

To further assess model generalizability, we evaluated the locked PSP and mPSP models in an independent multicenter validation cohort comprising 80 patients, including 40 with ESCC and 40 with EAC. Model performance was assessed using the area under the receiver operating characteristic curve (AUC), sensitivity, specificity, overall accuracy, and precision-recall (PR) analysis at the prespecified decision thresholds.

**Comparative analysis with published results**

We systematically identified relevant studies through PubMed searches (2020-2026) using the keywords “esophageal cancer early detection”. The included studies reported performance metrics (AUC, sensitivity, specificity) for EC detection and allowed direct extraction of the best performance values from results sections or tables. Although we aimed for a fair comparison, the test datasets and evaluation protocols differed across studies. The data are presented as reported in the original publications.

**Model evaluation and nested cross-validation**

To rigorously assess model generalization and avoid optimistic bias in high-dimensional omics data, we implemented a strict nested cross-validation framework using ‘scikit-learn’ library (version 1.7.2) in Python. The ED model used a 10 outer ×5 inner fold design, whereas the PSP and mPSP models used a 5 outer×5 inner fold design. In this dual-loop architecture, the dataset was first split into outer training and test sets. Within each outer training set, inner cross-validation was performed solely for hyperparameter tuning. The optimal hyperparameters were then used to train the model, which was evaluated on the corresponding outer test set. Importantly, oversampling, mutual information filtering, LASSO feature selection, and hyperparameter tuning were performed exclusively within each inner-loop training partition. Oversampling was never applied before data splitting or to the outer-loop test samples, thereby preventing information leakage.

**Detection of serum biomarkers**

Serum concentrations of CYFRA21-1, CEA, SCCA, and CA19-9 were quantified using electrochemiluminescence immunoassays. The established cut-off values were 3.3 ng/mL for CYFRA21-1, 5 ng/mL for CEA, 3 ng/mL for SCCA, and 27 U/mL for CA19-9. Patients were classified as biomarker-negative if levels of all four markers fell below their respective cut-offs, whereas those with at least one marker exceeding the threshold were classified as biomarker-positive.

**Principal component analysis**

Principal component analysis was performed on the fragmentomic features of the combined validation cohort using GraphPad Prism 9.5.1 (GraphPad Software). Through unsupervised dimensionality reduction, the first two principal components capturing the dominant variance of the features were extracted, and the resulting score plot intuitively visualized the overall distribution and clustering patterns of samples from each center, enabling a direct assessment of batch heterogeneity and overall consistency across multi-center data.

**Decision curve analysis**

Decision curve analysis was performed using the 'rmda' R package to identify precancerous lesions and differentiate pathological subtypes. Net benefit was calculated across a clinically relevant probability threshold range of 1% to 80% and compared against the default strategies of “treat-all” and “treat-none”.

**Precision-recall analysis**

Precision-recall analysis was conducted using the precision recall curve and average precision score functions implemented in the Python ‘scikit-learn’ library. For each classification task, PR curves were generated based on predicted probabilities. Model performance was quantified using the average precision score, which summarizes the precision-recall relationship across classification thresholds.

**Statistical Analysis**

Statistical analyses were performed using GraphPad Prism (version 9.5.1; GraphPad Software) and MedCalc (version 19.4). Nonparametric statistical tests were applied for group comparisons. The Friedman test was used to analyze differences in fragment size distribution across multiple related groups. Unpaired two-group comparisons were performed using the Wilcoxon rank-sum test. Comparisons among multiple independent groups were conducted using the Kruskal–Wallis H test, followed by Dunn’s post hoc test with Bonferroni correction. Spearman’s rank correlation coefficient was calculated to assess associations between variables. The Jonckheere-Terpstra test was employed to verify the trend consistency of ED scores with pathological severity. To address the sample imbalance between ESCC and EAC groups, permutation tests (10,000 iterations) were performed to estimate empirical *P* values and validate the robustness of the differential analysis results.

Diagnostic performance metrics, including sensitivity and specificity were calculated based on true-positive, true-negative, false-positive, and false-negative classifications. ROC curve analysis was used to evaluate the diagnostic performance of the EC detection model, with optimal cut-off values determined using Youden’s index in the training cohort. Sample size estimation was performed assuming 90% statistical power, a two-sided α level of 0.05, a β error of 0.10, and an anticipated area under the ROC curve (AUC) of 0.95. DeLong's test was used to compare the AUCs. All P values were two-tailed, and statistical significance was defined as *P* < 0.05.

**References**

[1].	Wang, S., et al., Early detection of colorectal cancer using aberrant circulating cell-free mitochondrial DNA fragmentomics. Gut, 2025. 74(6): p. 961-970.

[2].	Liu, Y., et al., Aberrant fragmentomic features of circulating cell-free mitochondrial DNA as novel biomarkers for multi-cancer detection. EMBO molecular medicine, 2024. 16(12): p. 3169-3183.

[3].	Dang, M., et al., Circulating cell-free mtDNA fragmentomics for early detection of gastric cancer and precancerous lesions. Cell reports. Medicine, 2026: p. 102825.
