# Supplementary material for: Noninvasive Detection and Subtyping of Esophageal Cancer by Circulating Cell‐Free mtDNA Fragmentomics: A Multi‐Center Validation Study
Source: Adv Sci (Weinh). 2026 Oct 28:e78377. Online ahead of print. doi: 10.1002/advs.78377 (PMC13715668; doi:10.1002/advs.78377)
Supplement: Supplementary file 2 [file ADVS-9999-e78377-s002.docx]

**Figure S1 | Demographic characteristics of study participants.** (A) Age (years) comparison of participants in the HC, BED, and EC groups. Dots represent individual participants; horizontal lines indicate the median and IQR. (B) Sex distribution of participants in each group, shown as proportions of male and female participants. ns, not significant; HC, healthy control; BED, benign esophageal diseases; EC, esophageal cancer; IQR, interquartile range.

**Figure S2 | Comparison of fragmentomic features by sex in the training cohort.** (A) Median ccf-mtDNA fragmentation profiles in the HC, BED, and EC groups, stratified by sex. Spearman’s correlation between male and female profiles is shown. (B) Principal component analysis of fragmentomic features within each group, colored by sex. HC, healthy control; BED, benign esophageal diseases; EC, esophageal cancer.

**Figure S3 | Comparison of fragmentomic features by age in the training cohort.** (A) Median ccf-mtDNA fragmentation profiles in the HC, BED, and EC groups, stratified by age. Spearman’s correlation between the two age-stratified profiles is shown. (B) Principal component analysis of fragmentomic features within each group, colored by age quartile (Age < Q1 *vs.* Age > Q3). Q1 and Q3 denote the 25th and 75th percentiles of age, respectively. HC, healthy control; BED, benign esophageal diseases; EC, esophageal cancer.

**Figure S4 | Comparison of fragmentomic features by smoking history in the training cohort.** (A) Median ccf-mtDNA fragmentation profiles in the HC, BED, and EC groups, stratified by smoking history (yes *vs.* no). Spearman’s correlation between the two smoking-stratified profiles is shown. (B) Principal component analysis of fragmentomic features within each group, colored by smoking history. HC, healthy control; BED, benign esophageal diseases; EC, esophageal cancer.

**Figure S5 | Comparison of fragmentomic features by alcohol history in the training cohort.** (A) Median ccf-mtDNA fragmentation profiles in the HC, BED, and EC groups, stratified by alcohol history (yes *vs.* no). Spearman’s correlation between the two alcohol-stratified profiles is shown. (B) Principal component analysis of fragmentomic features within each group, colored by alcohol history. HC, healthy control; BED, benign esophageal diseases; EC, esophageal cancer.

**Figure S6 | Comparison of fragmentomic features by BMI in the training cohort.** (A) Median ccf-mtDNA fragmentation profiles in the HC, BED, and EC groups, stratified by BMI (≤ 28 kg/m² *vs.* > 28 kg/m²). Spearman’s correlation between the two BMI-stratified profiles is shown. (B) Principal component analysis of fragmentomic features within each group, colored by BMI group. BMI, body mass index; HC, healthy control; BED, benign esophageal diseases; EC, esophageal cancer.

**Figure S7 | Comparison of fragmentation profile by tumor location in the training cohort.** (A) Median ccf-mtDNA fragmentation profiles in EC patients, stratified by tumor location (upper, middle, and lower). The inset shows pairwise Spearman’s correlation between the location-specific profiles. (B) Principal component analysis of fragmentomic features in EC patients, colored by tumor location. EC, esophageal cancer.

**Figure S8 | Comparison of fragmentomic features by serum biomarker status in the training cohort.** (A) Median ccf-mtDNA fragmentation profiles in EC patients, stratified by serum biomarker status (positive *vs.* negative). Spearman’s correlation between the positive and negative profiles is shown. (B) Principal component analysis of fragmentomic features in EC patients, colored by serum biomarker status. Serum biomarker-positive was defined as having at least one of the following markers above its established cut-off: CYFRA21-1, CEA, SCCA, or CA19-9; negativity was defined as all four markers below their respective cut-offs. EC, esophageal cancer.

**Figure S9 | Comparison of fragmentomic features across tumor stages in the EC training cohort.** (A) Median ccf-mtDNA fragmentation profiles in EC patients, stratified by tumor stage (early *vs.* advanced). Shaded areas indicate the IQR. Spearman’s correlation between the stage-specific median profiles is shown. (B) Comparison of correlation coefficients between individual fragmentation profiles and the median HC profile within each group. (C) Volcano plot of differential fragmentomic features between early-stage and advanced-stage. Each point represents one feature; dashed lines indicate the thresholds used to define differential fragmentomic features; positive log_2_ fold change indicates enrichment in early-stage EC. The two most enriched and two most depleted features were selected for the comparisons shown in panels D-G. (D-G) Comparison of four significantly differentiated features: (D) proportion of CAAA-end motifs, (E) proportion of CCG-end motifs, (F) AUDCneg in window 74, and (G) proportion of AGAT-end motifs. In panels B and D-G, dots represent individual participants; horizontal lines indicate the median and IQR. ns, not significant; *, *P* < 0.05; **, *P* < 0.01; ***, *P* < 0.001; IQR, interquartile range; HC, healthy control; EC, esophageal cancer; AUDCneg, Area Under the Distribution Curve of the negative peaks.

**Figure S10 | Comparison of fragmentomic features across tumor size categories in the EC training cohort.** (A) Median ccf-mtDNA fragmentation profiles in EC patients, stratified by tumor size (≤ 3 cm *vs.* > 3 cm). Shaded areas indicate the IQR. Spearman’s correlation between the size-stratified median profiles is shown. (B) Comparison of correlation coefficients between individual fragmentation profiles and the median HC profile within each group. (C) Volcano plot of differential fragmentomic features between tumor size ≤ 3 cm and > 3 cm. Each point represents one feature; dashed lines indicate the thresholds used to define differential fragmentomic features (as defined in Methods); positive log_2_ fold change indicates enrichment in tumor size > 3 cm. The two most enriched and two most depleted features were selected for the comparisons shown in panels D-G. (D-G) Comparison of four significantly differentiated features: (D) AUDCneg in window 58, (E) AUDCneg in window 79, (F) proportion of CCGA-end motifs, and (G) correlation in window 39. In panels B and D-G, dots represent individual participants; horizontal lines indicate the median and IQR. ns, not significant; *, *P* < 0.05; **, *P* < 0.01; ***, *P* < 0.001; IQR, interquartile range; HC, healthy control; EC, esophageal cancer; AUDCneg, Area Under the Distribution Curve of the negative peaks.

**Figure S11 | Principal component analysis (PCA) of the combined validation cohort in HC, BED, and EC groups. PCA score plots of samples from three centers in (A) HC, (B) BED, and (C) EC groups. Dots are colored by the originating center. PC1 and PC2 are shown with their respective explained variance. HC, healthy control; BED, benign esophageal diseases; EC, esophageal cancer.**

**Figure S12 | Plasma protein concentrations of DNASE1L3, DNASE1, DFFB, and ENDOG in HC, BED, and EC groups.** (A–D) Scatter plots showing plasma concentrations (ng/mL) of (A) DNASE1L3, (B) DNASE1, (C) DFFB, and (D) ENDOG in healthy controls (HC; n = 15), patients with benign esophageal diseases (BED; n = 15), and patients with esophageal cancer (EC; n = 30). Each dot represents an individual sample. Horizontal lines indicate the median, and error bars denote the IQR. *, *P* < 0.05; **, *P* < 0.01; ***, *P* < 0.001; ns, not statistically significant; DNASE1L3, deoxyribonuclease 1-like 3; DNASE1, deoxyribonuclease 1; DFFB, DNA fragmentation factor subunit beta; ENDOG, endonuclease G; HC, healthy control; BED, benign esophageal disease; EC, esophageal cancer; IQR, interquartile range.

**Figure S13 | Plasma protein concentrations of DNASE1L3, DNASE1, DFFB and ENDOG in EAC and ESCC groups.** (A-D) Scatter plots showing plasma concentrations (ng/mL) of (A) DNASE1L3, (B) DNASE1, (C) DFFB, and (D) ENDOG in patients with esophageal adenocarcinoma (EAC; n = 15) and esophageal squamous cell carcinoma (ESCC; n = 15). Each dot represents an individual sample. Horizontal lines indicate the median, and error bars denote the IQR. **, *P* < 0.01; ns, not statistically significant; DNASE1L3, deoxyribonuclease 1-like 3; DNASE1, deoxyribonuclease 1; DFFB, DNA fragmentation factor subunit beta; ENDOG, endonuclease G; EAC, esophageal adenocarcinoma; ESCC, esophageal squamous cell carcinoma; IQR, interquartile range.

**Figure S14 | Workflow of the EC detection model.** HC, healthy control; BED, benign esophageal diseases; EC, esophageal cancer; LASSO, Least Absolute Shrinkage and Selection Operator.

**Figure S15 | Performance of the ED model in the training cohort.** (A) **Comparison of ED scores** in the HC, BED, and EC groups. The cut-off is indicated by the dashed line. Dots represent individual participants; horizontal lines indicate the median and IQR. (B) Receiver operating characteristic curves showing model performance for distinguishing EC from HC, EC from BED, and EC from non-EC (HC and BED). (C) Specificity of HC and BED groups, and (D) sensitivity of EC group derived from the ED model at the optimal cut-off. In panels C-E, error bars represent 95% CI. (E) AUC values of the outer CV test set in the training cohort. ns, not significant; ***, *P* < 0.001; HC, healthy control; BED, benign esophageal diseases; EC, esophageal cancer; ED, esophageal cancer detection; IQR, interquartile range; CI, confidence interval; AUC, area under the curve; CV, cross-validation.

**Figure S16 | Clinical utility and predictive performance of the ED model. (A) Decision curve analysis comparing the net benefit of the ED model with the “treat‑all” and “treat‑none” strategies in the combined validation cohorts. (B) Precision‑recall curve of the ED model in the combined validation cohorts, with AP values. ED, esophageal cancer detection; AUC, area under the curve; CV, cross-validation; AP, average precision.**

**Figure S17 | Comparison of five previously reported methods for EC detection using published performance data.** HC, healthy control; EC, esophageal cancer; ESCC, esophageal squamous cell carcinoma; EAC, esophageal adenocarcinoma; AUC, area under the curve.

**Figure S18 | Specificity of the ED model in the other gastrointestinal malignancies cohort.** (A) ED score comparison in GC and CRC groups. Dots represent individual participants; horizontal lines indicate the median and IQR. The dotted horizontal line indicates the optimal diagnostic cut-off value of the ED score, determined by the maximum Youden index in the training cohort. (B) True negative and false positive rates of the ED model in distinguishing patients with EC from other gastrointestinal cancers. IQR, interquartile range; GC, gastric cancer; CRC, colorectal cancer; EC, esophageal cancer; ED, esophageal cancer detection.

**Figure S19 | Associations of demographic, lifestyle, and serum biomarker status with the ED score in the combined validation cohorts.** ED score comparisons in the HC (A-E), BED (F-J), and EC (K-O) groups, stratified by sex, age (≤ 50 years *vs.* > 50 years), smoking history, alcohol history, and BMI (≤ 28 kg/m² *vs.* > 28 kg/m²). (P) ED score comparison in EC patients stratified by serum biomarker status (positive *vs.* negative). Dots represent individual participants; horizontal lines indicate the median and IQR. (Q) Receiver operating characteristic curves for distinguishing EC from non-EC (HC and BED) in the combined validation cohorts, stratified by serum biomarker status. Serum biomarker positivity was defined as having at least one of CYFRA21-1, CEA, SCCA, or CA19-9 above its established cut-off, whereas negativity was defined as all four markers below their respective cut-offs. **ns, not significant;** HC, healthy control; BED, benign esophageal diseases; EC, esophageal cancer; ED, esophageal cancer detection; BMI, body mass index; IQR, interquartile range.

**Figure S20 | Workflow for sensitivity estimation using integrated serum biomarkers and performance comparison with the ED model and the combined strategy.** (A) Workflow integrating ccf-mtDNA fragmentomic feature analysis with serological biomarker detection. (B) Sensitivity comparisons for EC detection among the serum biomarker panel, the ED score, and the combined strategy; error bars represent 95% CI. **EC, esophageal cancer;** ED, esophageal cancer detection; **CI, confidence interval**.

**Figure S21 | Workflow of the pathological subtype prediction model.** LASSO, Least Absolute Shrinkage and Selection Operator; PSP, pathological subtype prediction; GERD, gastroesophageal reflux disease; mPSP, modified pathological subtype prediction.

**Figure S22 | Comparison of feature-selection strategies and model performance using the lambda.1se and lambda.min criteria.** (A) Comparison of the 20 highest-ranked ccf-mtDNA fragmentomic features selected under the lambda.1se and lambda.min criteria. The complete models retained 126 and 336 features, respectively. (B) Venn diagram showing the overlap between the top 20 ranked features from the two feature-selection strategies; 15 features were shared, whereas five were unique to each criterion. (C-D) Receiver operating characteristic (ROC) curves comparing the PSP models constructed using lambda.1se- and lambda.min-selected features in the (C) development cohort and (D) independent validation cohort. DeLong’s test was used to compare the corresponding AUCs. AUC, area under the curve; PSP, pathological subtype prediction; ROC, receiver operating characteristic; ccf-mtDNA, circulating cell-free mitochondrial DNA.

**Figure S23 |** Predictive performance of the mPSP model. (A) ROC curve of the mPSP model for distinguishing ESCC from EAC in the training cohort. (B) Confusion matrix of the mPSP model in the training cohort; numbers indicate correctly and incorrectly classified samples. (C) AUC values on the outer CV test set, error bars represent 95% CI. (D) Decision curve analysis of the mPSP model compared with treat-as-ESCC and treat-none strategies. (E) PR curves of the mPSP model on training cohort, with the AP value indicated. (F) ROC curve of the mPSP model in the validation cohort. (G) Confusion matrix of the mPSP model in the validation cohort. (H) PR curve of the mPSP model in the validation cohort, with the AP value indicated. mPSP, modified pathological subtype prediction; ROC, receiver operating characteristic; ESCC, esophageal squamous cell carcinoma; EAC, esophageal adenocarcinoma; AUC, area under the curve; CV, cross-validation; PR, precision-recall; AP, average precision.

**Figure S24 | Subgroup performance of the mPSP model. (A-D)** Receiver operating characteristic curves showing the performance of the mPSP model for distinguishing ESCC from EAC, stratified by (A) smoking history, (B) alcohol history, (C) BMI (≤ 28 kg/m² *vs.* > 28 kg/m²), and (D) GERD history. mPSP, modified pathological subtype prediction; **ESCC, esophageal squamous cell carcinoma; EAC, esophageal adenocarcinoma; BMI,** body mass index; GERD, gastroesophageal reflux disease.
