# Supplementary material for: Noninvasive Detection and Subtyping of Esophageal Cancer by Circulating Cell‐Free mtDNA Fragmentomics: A Multi‐Center Validation Study
Source: Adv Sci (Weinh). 2026 Oct 28:e78377. Online ahead of print. doi: 10.1002/advs.78377 (PMC13715668; doi:10.1002/advs.78377)
Supplement: Supplementary file 3 [file ADVS-9999-e78377-s003.docx]

**Supplementary Table 1. Demographic and clinicopathological characteristics.**

| **Characteristics** | **Tangdu Hospital of FMMU** | **Fudan University Shanghai  Cancer Center** | **Tumor Hospital Affiliated to Xinjiang Medical University** | ***P* values** | | |
| --- | --- | --- | --- | --- | --- | --- |
| **Total, n** | 745 | 386 | 129 |  | | |
| **Healthy control (n)** | 150 | 100 | 35 |  | | |
| Age, median years (range) | 57 (21-79) | 57 (24-77) | 53 (30-78) | 0.5808^a^ | | |
| Sex, n (%) |  |  |  | 0.7845^b^ | | |
| Male | 117 (78.00) | 75 (75.00) | 28 (80.00) |  | | |
| Female | 33 (22.00) | 25 (25.00) | 7 (20.00) |  | | |
| Smoking history, n (%) |  |  |  | 0.1696^b^ | | |
| Yes | 11 (7.33) | 12 (12.00) | 6 (17.14) |  | | |
| No | 139 (92.67) | 88 (88.00) | 29 (82.86) |  | | |
| Alcohol history, n (%) |  |  |  | 0.2048^b^ | | |
| Yes | 16 (10.67) | 17 (17.00) | 7 (20.00) |  | | |
| No | 134 (89.33) | 83 (83.00) | 28 (80.00) |  | | |
| BMI (kg/m^2^), n (%) |  |  |  | 0.1180^b^ | | |
| ≤ 28 | 120 (80.00) | 88 (88.00) | 26 (74.29) |  | | |
| > 28 | 30 (20.00) | 12 (12.00) | 9 (25.71) |  | | |
| **Benign esophageal disease (n)** | 137 | 100 | 35 |  | | |
| Age, median years (range) | 57 (24-79) | 56 (37-81) | 52.5 (19-76) | 0.7010^a^ | | |
| Sex, n (%) |  |  |  | 0.7750^b^ | | |
| Male | 106 (77.37) | 81 (81.00) | 27 (77.14) |  | | |
| Female | 31 (22.63) | 19 (19.00) | 8 (22.86) |  | | |
| Smoking history, n (%) |  |  |  | 0.5407^b^ | | |
| Yes | 17 (12.41) | 17 (17.00) | 4 (11.43) |  | | |
| No | 120 (87.59) | 83 (83.00) | 31 (88.57) |  | | |
| Alcohol history, n (%) |  |  |  | 0.5125^b^ | | |
| Yes | 25 (18.25) | 23 (23.00) | 9 (25.71) |  | | |
| No | 112 (81.75) | 77 (77.00) | 26 (74.29) |  | | |
| BMI (kg/m^2^), n (%) |  |  |  | 0.0991^b^ | | |
| ≤ 28 | 112 (81.75) | 91 (91.00) | 28 (80.00) |  | | |
| > 28 | 25 (18.25) | 9 (9.00) | 7 (20.00) |  | | |
| **Esophageal cancer (n)** | 418 | 186 | 59 |  | | |
| Age, median years (range) | 57 (23-83) | 56 (31-85) | 56 (27-76) | 0.7048^a^ | | |
| Sex, n (%) |  |  |  | 0.5851^b^ | | |
| Male | 327 (78.23) | 141 (75.81) | 43 (72.88) | | |  |
| Female | 91 (21.77) | 45 (24.19) | 16 (27.12) | | |  |
| Smoking history, n (%) |  |  |  | 0.2764^b^ | | |
| Yes | 130 (31.10) | 46 (24.73) | 18 (30.51) |  | | |
| No | 288 (68.90) | 140 (75.27) | 41 (69.49) |  | | |
| Alcohol history, n (%) |  |  |  | 0.0701^b^ | | |
| Yes | 99 (23.68) | 60 (32.26) | 18 (30.51) |  | | |
| No | 319 (76.32) | 126 (67.74) | 41 (69.49) |  | | |
| BMI (kg/m^2^), n (%) |  |  |  | 0.3614^b^ | | |
| ≤ 28 | 350 (83.73) | 153 (82.26) | 45 (76.27) |  | | |
| > 28 | 68 (16.27) | 33 (17.74) | 14 (23.73) |  | | |
| Pathological type, n (%) |  |  |  | 0.3062^b^ | | |
| ESCC | 364 (87.08) | 155 (83.33) | 48 (81.36) |  | | |
| EAC | 54 (12.92) | 31 (16.67) | 11 (18.64) |  | | |
| Tumor location, n (%) |  |  |  | 0.1214^b^ | | |
| Lower | 129 (30.86) | 54 (29.03) | 11 (18.64) |  | | |
| Middle | 218 (52.15) | 102 (54.83) | 31 (52.54) |  | | |
| Upper | 71 (16.99) | 30 (16.14) | 17 (28.82) |  | | |
| GERD history, n (%) |  |  |  | 0.0970^b^ | | |
| Yes | 15 (3.59) | 14 (7.53) | 4 (6.78) |  | | |
| No | 403 (96.41) | 172 (92.47) | 55 (93.22) |  | | |
| TNM stage, n (%) |  |  |  | 0.0895^b^ | | |
| Ⅰ | 62 (14.83) | 34 (18.28) | 12 (20.34) |  | | |
| II | 179 (42.82) | 73 (39.25) | 16 (27.12) |  | | |
| III | 114 (27.27) | 48 (25.81) | 14 (23.72) |  | | |
| IV | 63 (15.08) | 31 (16.66) | 17 (28.82) |  | | |
| Tumor size, n (%) |  |  |  | 0.4343^b^ | | |
| ≤ 3cm | 254 (60.77) | 115 (61.83) | 41 (69.49) |  | | |
| > 3cm | 164 (39.23) | 71 (38.17) | 18 (30.51) |  | | |
| **Precancerous lesion (n)** | 40 |  |  |  | | |
| Age, median years (range) | 57.5 (36-85) | / | / |  | | |
| Sex, n (%) |  |  |  |  | | |
| Male | 23 (57.50) | / | / | |  | |
| Female | 17 (42.50) | / | / | |  | |
| BMI (kg/m^2^), n (%) |  |  |  | |  | |
| ≤ 28 | 37 (92.50) | / | / | |  | |
| > 28 | 3 (7.50) | / | / | |  | |
| Tumor location,n (%) |  |  |  |  | | |
| Lower | 8 (20.00) | / | / |  | | |
| Middle | 21 (52.50) | / | / |  | | |
| Upper | 11 (27.50) | / | / |  | | |
| Stages, n (%) |  |  |  |  | | |
| LGIN | 8 (20.00) | / | / |  | | |
| HGIN | 32 (80.00) | / | / |  | | |

Abbreviation: FMMU, Fourth Military Medical University; BMI, body mass index; GERD, gastroesophageal reflux disease; TNM, tumor-node-metastasis; ESCC, esophageal squamous cell carcinoma; EAC, esophageal adenocarcinoma; LGIN, low-grade intraepithelial neoplasia; HGIN, high-grade intraepithelial neoplasia. ^a^ The *P* values were calculated using a Kruskal-Wallis test. ^b^ The *P* values were calculated using a Pearson’s Chi-Square test.

**Supplementary Table 2. Demographic and clinicopathological characteristics of patients with other gastrointestinal cancers.**

| **Characteristics** | **Tangdu Hospital of FMMU** |
| --- | --- |
|  |  |
| Age, median years (range) | 59 (38-84) |
| Sex, n (%) |  |
| Male | 52 (65.00) |
| Female | 28 (35.00) |
| Pathological type, n (%) |  |
| GC | 40 (50.00) |
| CRC | 40 (50.00) |

Abbreviation: FMMU, Fourth Military Medical University; GC, gastric cancer; CRC, colorectal cancer.

**Supplementary Table 3. Summary of ccf-mtDNA sequencing data.**

| **Case type** | **Number of cases** | **Average number of total reads** | **Average depth** | **Average Q30** |
| --- | --- | --- | --- | --- |
|  |  |  |  |  |
| Healthy control | 285 | 17,164,832 | 4,363 | 96.35% |
| Benign esophageal diseases | 272 | 18,246,917 | 4,061 | 95.59% |
| Esophageal cancer | 663 | 18,379,694 | 3,695 | 96.18% |
| Precancerous lesion | 40 | 17,393,607 | 3,939 | 96.74% |
| Colorectal cancer | 40 | 16,425,465 | 3,895 | 97.23% |
| Gastric cancer | 40 | 17,813,141 | 3,658 | 96.98% |
| Total | 1,340 | 17,989,675 | 3,924 | 96.17% |

| **Supplementary Table 4. Performance of EC detection model in three validation cohorts.** | | | | | | |
| --- | --- | --- | --- | --- | --- | --- |
|  | | **EC** | **non-EC** | **AUC (95% CI)** | **Sensitivity (95% CI)** | **Specificity (95% CI)** |
| **Internal validation cohort** | | | | | | |
| EC *vs.* HC | 118 | | 45 | 0.9944 (0.9876-1.0000) | 0.9407 (0.8826-0.9710) | 0.9778 (0.8843-0.9989) |
| EC *vs.* BED | 118 | | 45 | 0.9580 (0.9262-0.9898) | 0.9407 (0.8826-0.9710) | 0.8889 (0.7650-0.9516) |
| EC *vs.* non-EC | 118 | | 90 | 0.9762 (0.9585-0.9939) | 0.9407 (0.8826-0.9710) | 0.9333 (0.8621-0.9691) |
| **External validation cohort** | | | | | | |
| EC *vs.* HC | 150 | | 100 | 0.9812 (0.9691-0.9933) | 0.9333 (0.8816-0.9634) | 0.9100 (0.8377-0.9519) |
| EC *vs.* BED | 150 | | 100 | 0.9684 (0.9511-0.9856) | 0.9333 (0.8816-0.9634) | 0.8600 (0.7786-0.9147) |
| EC *vs.* non-EC | 150 | | 200 | 0.9748 (0.9623-0.9873) | 0.9333 (0.8816-0.9634) | 0.8850 (0.8334-0.9221) |
| **Chinese minority cohort** | | | | | | |
| EC *vs.* HC | 40 | | 35 | 0.9886 (0.9720-1.0000) | 0.9000 (0.7695-0.9604) | 0.9714 (0.8547-0.9985) |
| EC *vs.* BED | 40 | | 35 | 0.9450 (0.8884-1.0000) | 0.9000 (0.7695-0.9604) | 0.9143 (0.7762-0.9704) |
| EC *vs.* non-EC | 40 | | 70 | 0.9668 (0.9318-1.0000) | 0.9000 (0.7695-0.9604) | 0.9429 (0.8621-0.9776) |
| Abbreviation: HC, healthy control; BED, benign esophageal diseases; EC, esophageal cancer; AUC, area under the curve; CI, confidence interval. Note: non-EC stands for non-esophageal cancer and includes both healthy controls and patients with benign esophageal disease. | | | | | | |

| **Supplementary Table 5. Performance of EC detection model in the subtypes.** | | | | | |
| --- | --- | --- | --- | --- | --- |
|  | **EC** | **non-EC** | **AUC (95% CI)** | **Sensitivity (95% CI)** | **Specificity (95% CI)** |
| **Tumor stage** | | | | | |
| Early-stage *vs.* non-EC | 169 | 360 | 0.9687 (0.9549-0.9824) | 0.9290 (0.8800-0.9589) | 0.9083 (0.8741-0.9340) |
| Advanced-stage *vs.* non-EC | 139 | 360 | 0.9817 (0.9715-0.9918) | 0.9353 (0.8815-0.9656) | 0.9083 (0.8741-0.9340) |
| **Tumor location** | | | | | |
| Upper *vs.* non-EC | 49 | 360 | 0.9817 (0.9665-0.9969) | 0.9388 (0.8348-0.9790) | 0.9083 (0.8741-0.9340) |
| Middle *vs.* non-EC | 166 | 360 | 0.9721 (0.9589-0.9852) | 0.9217 (0.8706-0.9537) | 0.9083 (0.8741-0.9340) |
| Lower *vs.* non-EC | 93 | 360 | 0.9751 (0.9608-0.9895) | 0.9462 (0.8803-0.9768) | 0.9083 (0.8741-0.9340) |
| **Histological subtype** |  |  |  |  |  |
| ESCC *vs.* non-EC | 276 | 360 | 0.9752 (0.9654-0.9850) | 0.9275 (0.8907-0.9526) | 0.9083 (0.8741-0.9340) |
| EAC *vs.* non-EC | 32 | 360 | 0.9688 (0.9391-0.9984) | 0.9688 (0.8426-0.9984) | 0.9083 (0.8741-0.9340) |
| **Endoscopic diagnostic result of EEC** | | | | | |
| HGIN *vs*. non-EC | 29 | 360 | 0.9665 (0.9423-0.9908) | 0.9310 (0.7804-0.9877) | 0.9083 (0.8741-0.9340) |
| Cancer *vs.* non-EC | 140 | 360 | 0.9691 (0.9540-0.9842) | 0.9286 (0.8735-0.9607) | 0.9083 (0.8741-0.9340) |
| **Serum biomarker status** | | | | | |
| Positive *vs.* non-EC | 65 | 360 | 0.9826 (0.9718-0.9935) | 0.9692 (0.8946-0.9945) | 0.9083 (0.8741-0.9340) |
| Negative *vs.* non-EC | 243 | 360 | 0.9724 (0.9612-0.9836) | 0.9218 (0.8811-0.9494) | 0.9083 (0.8741-0.9340) |

Abbreviation: EC, esophageal cancer; ESCC, esophageal squamous cell carcinoma; EAC, esophageal adenocarcinoma; HGIN, high-grade intraepithelial neoplasia; EEC, early-stage EC; AUC, area under the curve; CI, confidence interval. Note: non-EC stands for non-esophageal cancer and includes both healthy controls and patients with benign esophageal disease.

**Supplementary Table 6. Performance of EC detection model in precancerous cohort.**

|  | **HGIN** | | **LGIN** | | **non-EC** | **AUC (95% CI)** |
| --- | --- | --- | --- | --- | --- | --- |
| HGIN *vs.* non-EC | | 32 | 0 | 90 | | 0.9597 (0.9243-0.9952) |
| LGIN *vs.* non-EC | | 0 | 8 | 90 | | 0.5917 (0.4355-0.7478) |

Abbreviation: EC, esophageal cancer; HGIN, high-grade intraepithelial neoplasia; LGIN, low-grade intraepithelial neoplasia; AUC, area under the curve; CI, confidence interval. Note: non-EC stands for non-esophageal cancer and includes both healthy controls and patients with benign esophageal diseases.

**Supplementary Table 7. Predictive performance of the PSP and mPSP models.**

|  | **AUC (95% CI)** | **Sensitivity (95% CI)** | **Specificity (95% CI)** |
| --- | --- | --- | --- |
| **PSP model** |  |  |  |
| Training cohort | 0.9522 (0.9297-0.9747) | 0.8994 (0.8708-0.9223) | 0.8750 (0.7637-0.9381) |
| Validation cohort | 0.9231 (0.8557-0.9905) | 0.8750 (0.7389-0.9454) | 0.9000 (0.7695-0.9604) |
| **mPSP model** |  |  |  |
| Training cohort | 0.9570 (0.9351-0.9788) | 0.9089 (0.8813-0.9306) | 0.8929 (0.7853-0.9500) |
| Validation cohort | 0.9406 (0.8767-1.0000) | 0.9500 (0.8350-0.9911) | 0.9000 (0.7695-0.9604) |

Abbreviation: PSP, pathological subtyping prediction; mPSP, modified PSP; AUC, area under the curve; CI, confidence interval. Sensitivity was calculated with esophageal squamous cell carcinoma as the positive class, and specificity with esophageal adenocarcinoma as the negative class.

**Supplementary Table 8. Multi-variable logistic regression analysis for differentiating ESCC and EAC.**

| **Variables** | **β** | **S.E.** | **Wald** | ***P*-value** |
| --- | --- | --- | --- | --- |
| Constant | 2.4910 | 0.4199 | 35.1861 | < 0.0001 |
| Cigarette smoking | -0.2552 | 0.6387 | 0.1597 | 0.6894 |
| Alcohol drinking | 0.2273 | 0.6934 | 0.1075 | 0.7430 |
| GERD history | -2.7924 | 0.9098 | 9.4202 | 0.0021 |
| BMI (kg/m^2^) | -0.3870 | 0.2374 | 2.6584 | 0.1030 |
| CEA (ng/mL) | -0.8055 | 0.3913 | 4.2381 | 0.0395 |
| CA19-9 | -0.2536 | 0.2842 | 0.7960 | 0.3723 |
| CYFRA21-1 | 0.1686 | 0.3568 | 0.2232 | 0.6366 |
| SCCA | 0.1979 | 0.3417 | 0.3356 | 0.5624 |
| PSP score | 0.9422 | 0.2694 | 12.2335 | 0.0005 |

Abbreviation: GERD, gastroesophageal reflux disease; BMI, body mass index; CEA, carcinoembryonic antigen; CA19-9, carbohydrate antigen 19-9; CYFRA21-1, cytokeratin 19 fragment; SCCA, squamous cell carcinoma antigen; AUC, area under the curve; PSP, pathological subtyping prediction; S.E., Standard Error.
